# An Overview of Various Applications of Cadmium Carboxylate Coordination Polymers

**DOI:** 10.3390/molecules29163874

**Published:** 2024-08-15

**Authors:** Gina Vasile Scaeteanu, Catalin Maxim, Mihaela Badea, Rodica Olar

**Affiliations:** 1 Department of Soil Sciences, Faculty of Agriculture, University of Agronomic Sciences and Veterinary Medicine, 59 Mărăști Str., 011464 Bucharest, Romania; gina.scaeteanu@agro-bucuresti.ro; 2 Department of Inorganic, Organic Chemistry, Biochemistry and Catalysis, Faculty of Chemistry, University of Bucharest, 90–92 Panduri Str., S5, 050663 Bucharest, Romania; catalin.maxim@chimie.unibuc.ro (C.M.); mihaela.badea@chimie.unibuc.ro (M.B.)

**Keywords:** cadmium, coordination polymer, carboxylate, sensor, storage

## Abstract

This review highlights the most recent applications of Cd(II)-carboxylate-based coordination polymers (Cd(II)-CBCPs), such as sensors, catalysts, and storage materials, in comparison with those of Zn(II) counterparts. A wide range of species with luminescence properties were designed by using proper organic fluorophores, especially a carboxylate bridging ligand combined with an ancillary N-donor species, both with a rigid structure. These characteristics, combined with the arrangement in Cd(II)-CBCPs’ structure and the intermolecular interaction, enable the sensing behavior of a plethora of various inorganic and organic pollutants. In addition, the Lewis acid behavior of Cd(II) was investigated either in developing valuable heterogeneous catalysts in acetalization, cyanosilylation, Henry or Strecker reactions, Knoevenagel condensation, or dyes or drug elimination from wastewater through photocatalysis. Furthermore, the pores structure of such derivatives induced the ability of some species to store gases or toxic dyes. Applications such as in herbicides, antibacterials, and electronic devices are also described together with their ability to generate nano-CdO species.

## 1. Introduction

Cd(II) complexes similar to Zn(II) might seem unattractive to researchers due to their diamagnetism.

Similarly, a comparison between these ions involving their relationship with living organisms indicates that zinc is an essential element while cadmium is recognized for its toxicity. Cd(II) toxicity is well documented: WHO includes it in the list of the most dangerous chemicals and the International Agency for Research on Cancer lists it as a group 1b carcinogen [1,2]. Data indicate its accumulation in the human body from the food chain [3] and water sources [4], breathing, smoking and exposure to particles resulting from ore exploitation and processing [5,6]. Some human activities such as combustion of fossil fuels, waste, and metal ores are also sources of the spread the cadmium into environment [4] from where it can be also retained by humans over time. Fortunately, cadmium toxicity and its adverse effects on the human body together with its elimination through chelation therapy are well documented [4,5,6,7,8,9].

On the other hand, similar to Zn(II), Cd(II) exhibits a good Lewis acid activity that allows its involvement in several catalytic processes. But, despite the continuous interest in the structural aspects of Cd(II) species, research contributions describing such applications are currently limited due to its toxicity [10]. Moreover, its versatile coordination behavior generates valuable applications as sensors based on luminescent properties for species with properly selected ligands [11,12]. Furthermore, its ability to adopt various stereochemistry allows for a diverse structural arrangement in the network, which generates pores and thus induces the ability of some materials to store gaseous or solid species similar to the Zn(II) counterpart [13].

**Figure 1:**
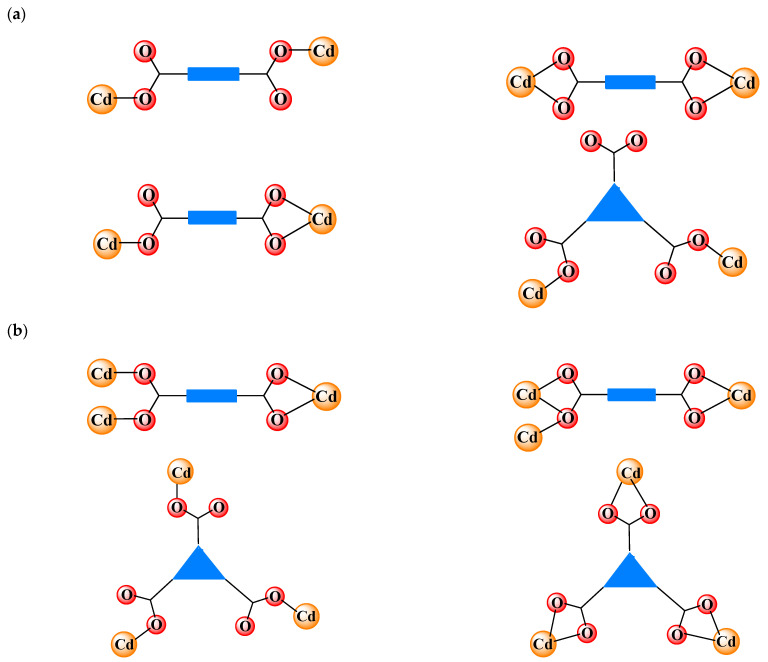

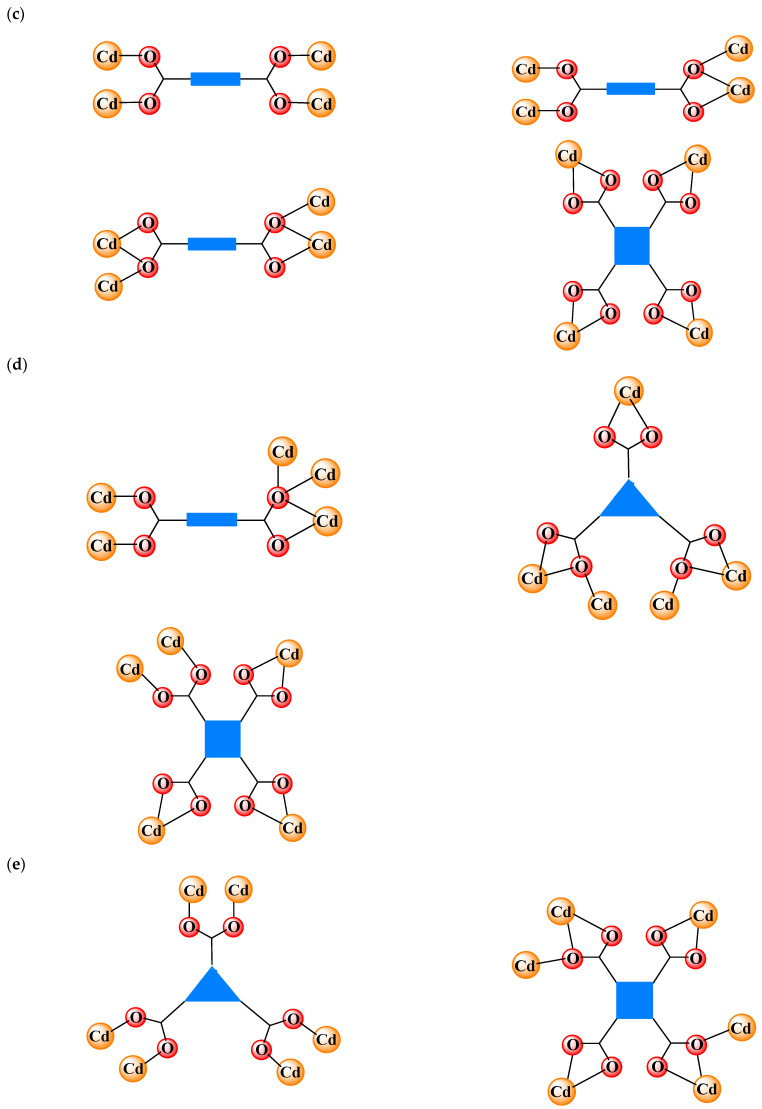

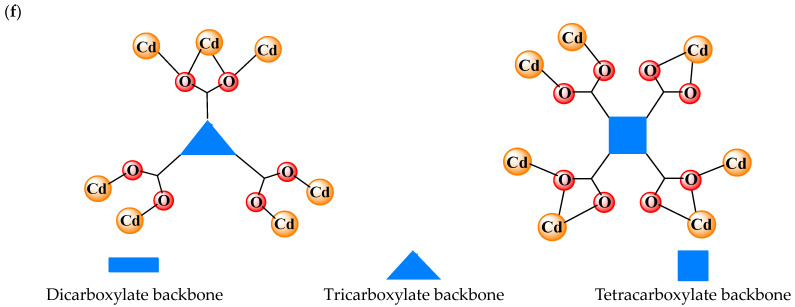
Coordination modes of the carboxylate group in Cd(II)-carboxylate-based coordination polymers: bridge between (**a**) two, (**b**) three, (**c**) four, (**d**) five, (**e**) six, and (**f**) seven ions.

Some of these properties are characteristic of a polymeric structure, which can be achieved by a suitable molar ratio and by mixing a carboxylate derivative with another appropriate bridging ligand. Usually, polycarboxylate derivatives possess an enhanced ability to generate two- or three-atom connections between metallic ions in coordination polymer (CP) structures (Figure 1) [11,14]. 

As can be seen from Figure 1, besides the most common two- or three-atom bridges, other bridges of up to seven atoms connected in the network structure have also been reported. It is worth mentioning that in some complex structures, two or three different coordination modes of the bridging carboxylate group may even appear. Hence, generally, several aromatic and heterocyclic two-, three- or tetracarboxylate derivatives have usually been used for the synthesis of Cd(II)-carboxylate-based coordination polymers (Cd(II)-CBCPs) with diverse structures and properties. 

A burgeoning class of adsorbent materials is represented by metal–organic frameworks (MOFs), species with desirable attributes such as a tunable crystal structure and morphology, ultrahigh specific surface area and porosity, adaptable surface chemistry, high thermal stability, open metal sites, and high degree of crystallinity and robustness [15,16]. They also exhibit high compatibility with other materials leading to composites with valuable physical and chemical properties [17]. Current efforts in the field are centered on enhancing the specific surface area and the gas-binding affinity, both favored by Cd(II) cations due to their large volume.

Conversely, by comparison with other CBCPs, it is rather difficult to control both morphology and structure [16] as well as create a mixture either of proper organic linkers (carboxylate and a N-donor ligand) or to combine Cd(II) with another cation in the structure of this kind of species. 

On the other hand, Cd(II), similar to Zn(II), shows advantages in developing CPs. These advantages are supported by its d^10^ configuration that allows for a flexible stereochemistry and consequently for its geometries to accommodate ligand parameters easily. It varies from tetrahedral stereochemistry through trigonal, bipyramidal, and square pyramidal to octahedral stereochemistry, adopting different distortion degrees. The stereochemistry control is achieved by a proper selection of either ligands or molar ratio.

A literature survey evidenced that some carboxylate derivatives, di- and three-carboxylate benzene derivatives with a rigid backbone, exhibit the ability to develop polymeric structures by coordination with Cd(II). Their packing features can then be controlled through polydentate species acting as bridges, either a carboxylate alone or in combination with another ligand. These supplementary ligands are usually heterocycle N-based derivatives, such as pyridine, thiazole, imidazole, bipyridine, phenanthroline, or a combination of such derivatives. Besides modulation of both topology and porosity, these species can also provide an additional fluorophore source into the complex.

**Scheme 1:**
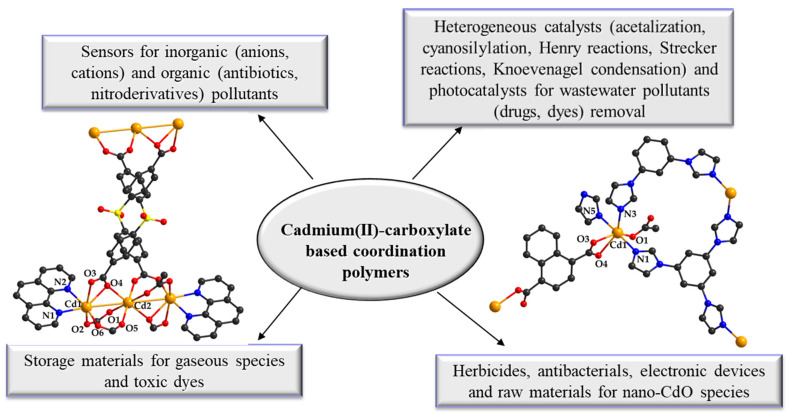
Applications of cadmium (II)–carboxylate-based coordination polymers.

As a result of the rich structural chemistry of Cd(II)-CBCPs, these compounds exhibit potential applications as sensors for an inorganic or organic compound [12,15], materials for gas storage or separation [17,18,19], a heterogeneous catalyst [17], electronic devices [20,21], herbicides, and raw materials for the nano-CdO generation (Scheme 1). 

The most recent data on the above-mentioned applications are summarized by categories in the following sections.

Once the main objectives of this review paper were established, a literature review was conducted to identify the most relevant papers that address our objectives. For this purpose, databases were used, such as Clarivate Analytics’ Web of Science (WoS), Elsevier’s Scopus (Scopus), and Science Direct, for searching papers. The following keywords and/or combinations of keywords have been used: “cadmium coordination polymer”, “cadmium” + “carboxylate ligand”, “cadmium” + “carboxylate complexes” + “luminescence”/”sensor”/”storage”/”catalyst”/“adsorbent”/”properties”. This procedure helped identify several categories of papers (reviews, reports, communications), from which the suitable ones have been selected for this review, and classified using properties and applications as criteria. Furthermore, the reference lists of these papers were scanned to identify further related papers. As a result, the database obtained was enriched with additional papers discovered with the snowballing technique applied to initially classified papers. All the classified papers were used for extracting data that was further included in the review paper, emphasizing the applicability of cadmium carboxylate coordination polymers.

As today’s area of coordination polymers is vast and developing fast, it was considered suitable that this review includes papers published in the last decade.

## 2. Basic Applications of Cadmium (II) Carboxylate Coordination Polymers

The Cd(II) species described below either exhibit fluorescence or possess stable and robust framework structures with a proper porosity that allows retention, sometime selective, of small guest molecules like gases, dyes, or drugs. Based on photocatalytic properties, some Cd(II)-CBCPs were also studied as potential agents able to remove dyes or drugs from wastewater, while additional applications include their use as herbicides, antibacterial species, as drugs in uterine fibrosis treatment, and in electronic devices like gate dielectrics, proton conductive materials, or cathodes in Li–Se batteries. Special attention is also paid to the use of such CPs for the nano-CdO generation, another material with valuable uses is several fields. 

Table 1 presents examples of Cd(II)-carboxylate-based coordination polymers with diverse applications as well as the ligands’ nature, synthesis method, and characteristics.

### 2.1. Sensors Based on Cadmium (II) Carboxylate Coordination Polymers

Some water pollutants are harmful for both humans and the environment. These species are the result of industrial, hospital, and domestic activities; they are antibiotics, dyes, and nitro-derivatives, as well as cationic and anionic inorganic harmful compounds [22]. Hence, their monitoring requires both sensitive and selective species such as fluorescent Zn(II) and Cd(II) complexes [12,15]. Similar to Zn(II) species, the luminescence of Cd(II)-CBCPs can be related to π-electron-rich fluorescent ligands. The luminescence in these cases can originate from intra-ligand (IL), ligand-to-ligand (LLCT), or metal-to-ligand (MLCT) spin-allowed charge-transfer processes [11,12]. The role of a fluorescent ligand can be played by the carboxylate linker, the supplementary N-donor ligand, or both. Since d^10^ ions such as Zn(II) and Cd(II) have the ability to regulate the emission wavelength of organic materials, several species based on benzene polycarboxylate rigid tectons were developed as luminescent materials [21,22,23,24,25,26,27,28,29,30,31,32,33,34,35,36,37,38,39,40,41,42,43,44], some of them with the ability to selectively detect certain organic or inorganic species. 

**Figure 2:**
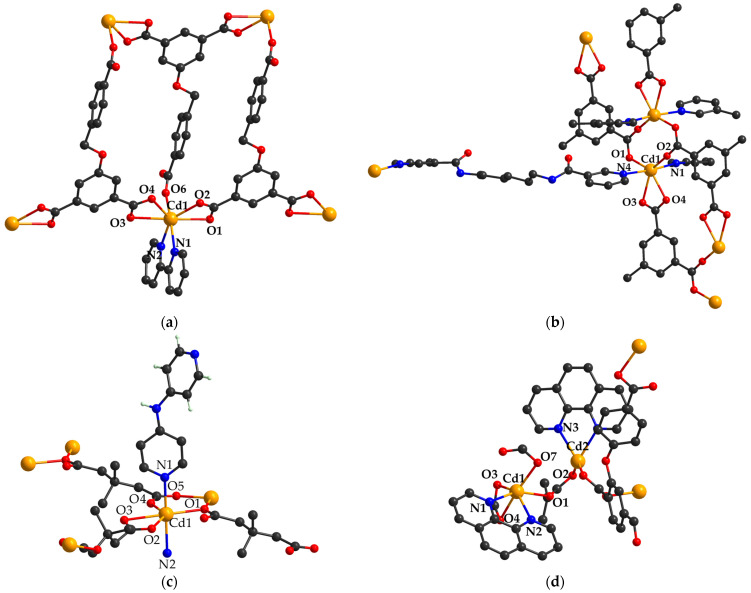
The coordinative site of complexes (**1**) (**a**), (**2**) (**b**), (**13**) (**c**), and (**17**) (**d**) (adapted from refs. [45,46,55,59]).

Among these, [Cd(bpmta)_0.5_(1,2-bdc)(H_2_O)]_n_ (**1**) (H_2_bdc = benzenedicarboxylic acid; bpmta = N,N′-bis(pyridin-3-ylmethyl)-terephthalamide) (Figure 2a) prepared under hydrothermal conditions shows multi-functional fluorescence responses towards Fe(III), CrO_4_^2−^, Cr_2_O_7_^2−^ and 2,6-dichloro-4-nitroaniline (2,6-DC-4-NA) as well as a good stability within a wide range of pH values [45].

The sensing of nitroaromatic compounds in aqueous media is an important aspect for the protection of both the environment and human health. For this purpose, Cd(II) CP [Cd_3_(bpy)_3_(cia)_2_]_n_ (**2**) was characterized as a one-dimensional ladder-like chain based on 5-((4-carboxybenzyl) oxy) isophthalic acid (5-H_3_cia) and 2,2′-bipyridine (2,2′-bpy) (Figure 2b). The compound detects nitrobenzene (NBZ) in an aqueous solution with high sensitivity and selectivity, with a limit of detection (LOD) of 3.03 × 10^−9^ M. The fluorescence-quenching mechanism consists of NB absorption in the pore, thus blocking thus the LLCT [46].

The MOF {[Cd(H_4_pbitb)]⋅2DMF⋅8H_2_O}_n_ (**3**) (H_6_pbitb = 4,4′,4″,4‴-(1,4-phenylenebis (1H-imidazole-2,4,5- triyl))tetrabenzoic acid; DMF = *N*,*N*′-dimethylformamide) was successfully constructed under solvothermal conditions. The structural analysis revealed a 3D framework and exhibited good stability in aqueous solutions within the pH range between 4 and 12. Its intense luminescence emission could be used to quickly and sensitively detect Fe(III), MnO_4_^−^, and 2,4,6-trinitrophenol (TNP) in aqueous solutions with a high quenching constant and a low detection limit, even in the presence of other competitor ions [47].

Compounds {[Cd_2_(1,3-bdc)_2_(H_2_O)_4_(hdn)]_2_H_2_O}_n_ (**4**) and {[Cd(mbdc)(hdn)]H_2_O}_n_ (**5**) (H_2_mbdc = 5-methy-1,3-benzenedicarboxylic acid; hdn = *N*,*N*′-(hexane-1,6-diyl)dinicotinamide) adopt an interesting topology in the solid state. Based on their luminescent properties, both compounds exhibit a good ability to detect NBZ in an ethanol suspension [48].

The CP [Cd(bzimip)(DMF)]_n_ (**6**), (H_2_bzimip = 5-(benzimidazole-1-yl)isophthalic) synthesized by the same method consists of a 3D network generated through a [CdO_5_N]_2_ cluster. The compound exhibits strong fluorescent properties, which enables it to detect antibiotics such as sulfathiazole (STZ), nitrofurazone (NFZ), and sulfadiazine (SDZ) by fluorescence quenching in an aqueous solution [49].

The excellent fluorescence performance for species [Cd_4_(Hbdcpb)_2_(H_2_O)_5_]_n_ (**7**) and {[Cd_2_(Hbdcpb)(2,2′-bpy)_2_(H_2_O)]·6H_2_O}_n_ (**8**) (H_5_bdcpb = 2,3-bis(3,5-dicarboxylphenxoy)benzoic acid) induces their sensing abilities for antibiotics. The results show that CP (**7**) exhibits high sensitivity in detecting SDZ and selectivity for nitrofurantoin (NFT) [50].

Another CP with formula [Cd_4_(2.2′-bpy)_4_(Hddpb)_2_·H_2_O]_n_ (**9**) (H_5_ddpb = 2,5ʹ-di(3,5-dicarboylphenoxy)benzoic acid) exhibits sensitive and selective detection for NFZ with a quenching efficiency (Ksv) of 1.08 × 10^3^ M^−1^ and LOD of 1.38 ppm [51].

The MOF {[Cd_2_(btdb)_2_(4,4′-bpy)]·DMF}*_n_* (**10**) (H_2_btdb = 4,4′-(benzo[*c*][1,2,5]thiadiazole-4,7-diyl)dibenzoic acid, 4,4′-bpy = 4,4′-bipyridine) structure is formed by a tetranuclear cluster based on a six-connected topological network. This behaves as a highly selective and sensitive luminescent sensor for L-histidine (His) by both turn-on and fluorescence blue-shift effects [52].

Compound [Cd(cphi)(Hbpz)]_n_ (**11**) (H_3_cphi = 5-(4-carboxyphenoxy)isopthalic acid; Hbpz = 3,3′5,5′-tetramethyl-4,4′-bipyrazole) behaves as a highly sensitive sensor for Fe(III) detection, through the decrease in its luminescence intensity in the presence of active sites [53]. Also, the solvothermal reaction allowed CP [Cd_2_(dtta)_2_]_n_ (**12**) (H_2_dtta = 2,5-di(1H-1,2,4-triazol-1-yl)terephthalic acid) to display an unusual 3D network. In addition, luminescent investigations suggested that (**12**) exhibits highly selective and sensitive detection for Cu(II) over other cations with a high Ksv value of 8.49 × 10^3^ M^−1^ [54].

The CP [Cd(3,3′-dmg)(dpam)]_n_ (**13**) (H_2_dmg = dimethyl glutaric acid, dpam = 4,4′-dipyridylamine) adopts a 2-fold interpenetrated 3D topology based on dimer-based layers pillared by an N-donor ligand (Figure 2c). The luminescent behavior is assigned to intra-ligand transitions in dpam aromatic rings and enables the NBZ detection in an ethanol suspension [55].

The compound [Cd(4-tkpvb)(5-*tert*-bipa)]_n_ (**14**) (4-tkpvb = 1,2,4,5-tetrakis(4-pyridylvinyl)benzene; 5-*tert*-H_2_bipa = 5-tert-butylisophthalic acid) behaves as an uncommon multi-responsive luminescent sensor for Hg(II), CrO_4_^2−^, and Cr_2_O_7_^2−^ ions in water with the detection limits of 0.15, 0.08, and 0.12 μM, respectively. The luminescence-quenching mechanism involves either the interaction of Hg(II) with free pyridyl groups or the overlap between the absorption band of anions and the excitation and/or emission bands of complex [56].

The 2D MOF [Cd(dpttz)(oba)]_n_ (**15**) (dpttz = 2,5-di(pyridine-4-yl)thiazolo[5,4-*d*]thiazole; H_2_oba = 4,4′-oxybis(benzoic acid)) shows an excellent luminescence property and a good stability in water. Moreover, this compound has remarkable sensitivity and selectivity for the detection of 4-nitroaniline (4-NA) and the CrO_4_^2−^ anion, with a low-limit detection of 0.52 and 1.37 μM. The fluorescence-quenching mechanism consists of the collapse of the framework, which generates the photoinduced electron transfer, and the resonance energy transfer [57].

The detection of Cu(II) and Ni(II) cations in an aqueous medium was investigated for [Cd_2_(btc)_2_(phen)_2_(H_2_O)_2_]_n_ (**16**) (H_3_btc = 1,3,5-benzentricarboxylic acid; phen = 1,10-phenantroline) based on their fluorescence properties. For both cations, the detection is achieved with a fast response, a wide linear range, and a very low detection limit. These features allow the use of CP for real water samples without any matrix interference [58].

A porous MOF [Cd_3_(cpota)_2_(phen)_3_]_n_·5nH_2_O (**17**) (H_3_cpota = 2-(4-carboxyphenoxy)terephthalic acid; phen = 1,10-phenanthroline) has been synthesized and characterized with an uncommon 3D microporous structure bearing [Cd_3_(phen)_3_(μ_2_-COO)_4_]^2+^ as building units (Figure 2d). This compound is an excellent probe for volatile organic ketones (acetone/2-butanone) and chromate/dichromate in an aqueous solution. The luminescence investigations in the aqueous solution at pH = 9 reveal that the species can efficiently and selectively detect Cr(VI) ions without the interference of other cations [59].

In order to obtain species with multiple targets sensing, the CP {[Cd_2_(edda)(phen)_2_]∙H_2_O}_n_ (**18**) (H_4_edda = 5,5′(ethane-1,2-diylbis(oxy)) diisophthalic acid) was designed and prepared under hydrothermal conditions. The structural analysis indicates that (**18**) possesses a 3D supramolecular framework via π–π interactions between phen molecules and it is tolerant at a wide range of pH values (2–13). Furthermore, the complex exhibits highly selective and sensitive fluorescence responses toward MnO_4_^−^, Cr(VI) ions, acetyl acetone (Hacac), and ascorbic acid (AA) by fluorescence quenching in the aqueous phase. The LODs reached the μM level for MnO_4_^−^ and Cr(VI) ions, nM for AA, and ppm for Hacac detection [60].

Another Cd(II) CP [Cd_3_(dcpb)_2_(datrz)(H_2_O)_3_]_n_ (**19**) was synthesized by combining the 4-(2′,3′-dicarboxylphenoxy) benzoic acid (H_3_dcpb) with the auxiliary ligand 3,5-diamino-1,2,4-triazole (datrz). The structure analysis shows a 2D layer structure. Further investigations reveal both the nature of water molecules and their thermal stability. The fluorescent explorations indicate a blue light emission and the ability to detect both the hypochlorite anion (ClO^−^) and the Hacac with a LOD of 0.18 and 0.056 μM, respectively [61].

The complex [Cd(bibt)(3,4-tdc)]_n_ (**20**) (bibt = 4,7-bi(1H-imidazol-1-yl)benzo-[2,1,3]thiadiazole; H_2_tdc = thiophene-dicarboxylic acid) synthesized under solvothermal conditions adopts a 2D structure with sql topology. The compound exhibits excellent stability in aqueous solutions within a pH range of 2 to 13. Furthermore, the compound selectively detects salicylaldehyde (SA) with a LOD of 0.087 μM. In addition, a portable fluorescent film and a light-emitting diode lamp based on (**18**) were further developed for the detection of SA [62].

The ultra-stable CP {[Cd_2_(ddb)(Hbimb)]∙3H_2_O}_n_ (**21**) (Hddb = 3,5-di(2′,4′-dicarboxylphenyl)benzoic acid; Hbimb = *ortho*-bis(imidazole-1-ylmethyl)benzene) adopts an unique 3D framework that consists of a tetranuclear {Cd_4_(COO)_8_} cluster and organic linkers rich in π-electrons. This species also exhibits an excellent luminescence-sensing ability towards nitrofuran (NF) in an aqueous medium. The low detection limit is accompanied by a high sensitivity and selectivity, and a recyclable behavior [63]. 

Therefore, a 3D Cd(II) CPs constructed from 1,3,5-tris(1-imidazolyl)benzene (tib) and several phenylenediacetic acid (H_2_pda) derivatives, {[Cd(1,2-pda)(tib)]·H_2_O}_n_ (**22**), [Cd_4_(1,3-pda)_4_(tib)_2_(dib)_2_]_n_·7nH_2_O (23) (dib = 1,3-bis(1-imidazolyl)benzene), and [Cd(1,4-pda)(tib)]_n_·4nH_2_O (24), have proven their effectiveness in tetracycline detection (TEC) [64].

Also, complexes [Cd(2,5-tdc)(tbb)]_n_ (**25**) (tbb = 1,4-bis(thiabendazole-1-yl)-2-butene) and {[Cd(tpca)(tbb)]·H_2_O}_n_ (**26**) (H_2_tpca = 2,3,5,6-tetrabromoterephtalic acid; tbb = 1,4-bis(thiabendazole-1-yl)-2-butene) have the ability to detect Fe(III), Hacac, and norfloxacin (NOR) [65].

A species based on the tricarboxylato ligand biphenyl-2,4′,5-tricarboxylic acid (H_3_bptc), namely [Cd_3_(bptc)_2_(H_2_O)_4_]_n_ (**27**), showed high quenching efficiency for testing acetone and Fe(III) ions in aqueous solution [66].

The MOF {[Cd(dint)(1,4-bdc)(H_2_O)]·ACN}_n_ (**28**) (dint = 1,4-di(imidazol-1-yl)naphthalene, ACN = acetonitrile) adopts a 4-fold interpenetrated network. This species behaves as a bis-color excited fluorescent sensor with a high sensitivity to vitamin B2. Interestingly, at an excitation wavelength of 230 nm, this was significantly quenched by a pesticide called nitenpyram, while at an excitation wavelength of 290 nm, the highest fluorescence quenching was achieved for imidacloprid [67].

A 2D MOF, [Cd(pddb)H_2_O]_n_ (**29**) (H_2_pddb = 4,4′-(pyridine-2,6-diyl)-dibenzoic acid), exhibits fascinating one-dimensional in-plane channels functionalized with active pyridine-N sites, and an excellent water and chemical stability. Furthermore, a species with a dual-emissive ratiometric fluorescent sensor for 2-(2-methoxyethoxy) acetic acid (MEAA) was obtained by Eu(III) functionalization. This aspect is important because MEAA is a metabolite of 2-(2-methoxyethoxy)ethanol, which affects the DNA and, as a result, exhibits teratogenic and toxic effects [68]. 

Some CPs with a 3D interesting topology, {Cd_3_(btc)_2_(btd-bpy)_2_]·1.5MeOH·4H_2_O}_n_ (**30**) and [Cd_2_(1,4-ndc)_2_(btd-bpy)_2_]_n_ (**31**) (btd-bpy = bis(pyridin-4-yl)benzothiadiazole; 1,4-ndc = naphthalene-1,4-dicarboxylic acid; MeOH = methanol), were hydro(solvo)thermal synthesized. Both species exhibit a fluorescence-quenching effect in the presence of Ag(I) as result of framework collapse, and an enhancement response for Al(III) and Cr(III), due to weak ion–framework interactions. Compound recognition occurs in water suspension with both high selectivity and sensitivity in a micromolar LOD [69].

To conclude, in order to obtain Cd(II)-CBCPs with a broad range of emission energy, the selection of multifunctional organic ligands is of utmost importance. Data indicated that the polycarboxylate ligands, especially those derived from rigid aromatic derivatives with both a large π-conjugated backbone and the ability to act as bridge, are excellent candidates to design such fluorescent-based materials. The properties can be further modulated by the appropriate selection of a second N-donor ligand (i.e., pyridine, imidazole, thiadiazole derivative) exhibiting the same characteristics as carboxylate ligands. 

The fluorescence quenching by inorganic or organic species with a volume that matches the pore dimensions enables their specific recognition and thus the possibility to develop sensors that may be used in the monitoring process of pollutants.

The Cd-CBCPs presented have proven their efficacy for the detection of different drugs and bioactive species (STZ, NFZ, SDZ, NFT, TEC, NOR, AA), cations (Fe(III), Cu(II), Ni(II)), anionic species (CrO_4_^2−^, Cr_2_O_7_^2−^, MnO_4_^−^), and organic pollutants (ketones, nitroaromatic derivatives).

All compounds described above as sensors are neutral species that exhibit a robust network, being both water and pH stable. Also, these share a common characteristic of the excitation wavelength in the UV domain, considering the fact that this comes from π→π* or n→π* transitions of the ligands, usually the N-based ancillary one.

An analysis of species described in this section indicates that the sensing ability of Cd-CBCPs comes from a correlation between fluorescence quenching and architecture collapse in interaction with a given analyte. For the majority of these species, the former process can be assigned either to the photoinduced electron transfer (PET) or to the inner filter effect (IFE). The PET effect is involved in quenching when the Cd-CBCP material has a LUMO with a higher energy than the analyte, and as a result, the electron transfer from the fluorescent material to the analyte occurs. When the excitation radiation of the fluorescent species is absorbed by the analyte, then the IFE mechanism occurs. Obviously, a combination of these two mechanisms is also possible.

### 2.2. Catalysts Based on Cadmium (II) Carboxylate Coordination Polymers

It has been proven that cadmium (II) centers present good catalytic performances based on their Lewis acid ability. Also, the identification of heterogeneous catalysts derived from CPs is a topic of great interest. These catalysts are more desirable than the homogeneous ones, for example, due to their easy recovery. From this perspective, the efficacy of CPs is due to their superiority over other materials, since CPs present a high surface area, large pores, and a high number of Lewis acid centers [70].

**Figure 3:**
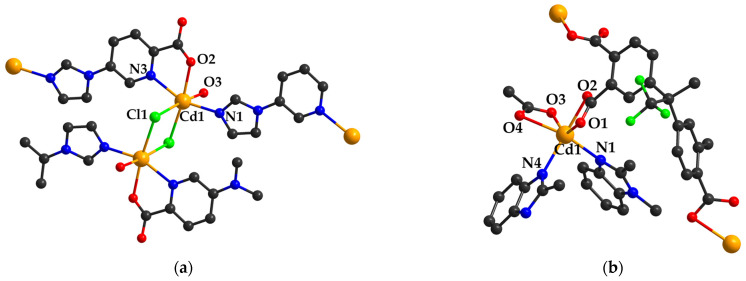

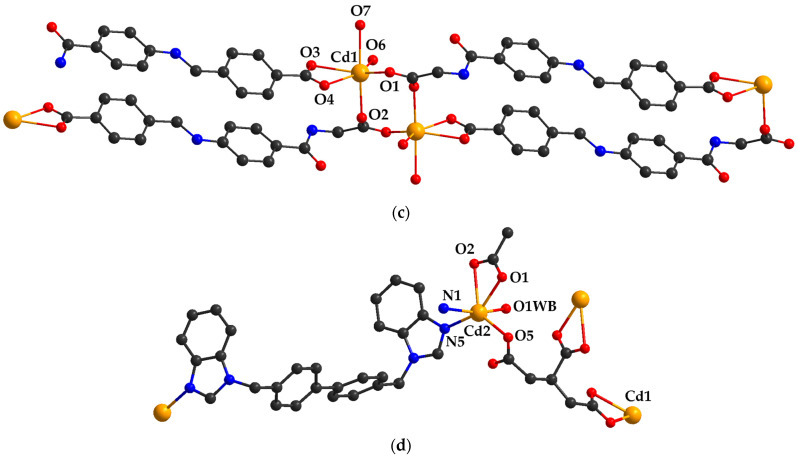
The coordinative site of complexes (**32**) (**a**), (**46**) (**b**), (**42**) (**c**), and (**50**) (**d**) (adapted from refs. [71,81,85,88]).

Consequently, CP formulated as [Cd(ipc)(Cl)(H_2_O)]_n_ (**32**) (Hipc = 5-imidazol-1-yl)-2-pyridine carboxylic acid) (Figure 3a) was evaluated for its catalytic activity in the acetalization reaction and the results indicated that it is an efficient Lewis acid catalyst. As the lifetime and reusability of catalysts are important indicators for their efficient use, the complex (**32**) was tested from these perspectives and it has given a 78, 81, and 82% yield for the second, third, and fourth runs, respectively. This catalyst can be readily recovered after being processed and reused [71].

Good Lewis catalytic activity of complex {[Cd(cbdcp)(H_2_O)_4_]∙(H_2_O)}_n_ (**33**) (H_3_cbdcpBr = 1-(3,5-dicarboxybenzyl)-4,4′-bipyridinium bromide) in a cyanosilylation reaction has been demonstrated by a high yield of the product obtained (93%), easy recovery, and the potential for reuse. Although the authors in [72] stated that it is possible to determine the catalytic activity by the cadmium ions with coordinated water molecules or a pyridinium unit, the catalytic centers of the cyanosilylation reaction are subjected to further studies.

Another CP formulated, [Cd_2_(1,4-ndc)_2_(DMF)_2_]_n_ (**34**), was reported [73] due to catalytic activity for a cyanosilylation reaction of aromatic aldehydes with nitro substituents in different positions. This was the result of the presence of micropores inside the structure after partial dissociation of DMF molecules. 

The CPs [Cd(3,3′-dbdc)(2,2′-bpy)(H_2_O)]_n_ (**35**) (H_4_(3,3′-dbdc) = 3,3′-dihydroxy-(1,1′-biphenyl)-4,4′-dicarboxylic acid) and [Cd(4,4′-dbdc)(phen)]_n_ (**36**) (H_4_(4,4′-dbdc) = 4,4″-dihydroxy-(1,1′-biphenyl)-3,3′-dicarboxylic acid) were tested as heterogeneous catalysts for the Henry reaction [74]. According to the literature [75], the Henry reaction, also known as the nitroaldol reaction, is a valuable tool used for C–C bond formation and the resultant product (nitroaldol) may lead to different oxygen- and nitrogen-containing derivatives. Also, the Henry reaction requires catalysts and many studies are investigating what the appropriate catalyst or catalytic system is. As for CPs (**35**) and (**36**), the catalytic activity was explored in the transformations of various aldehyde substrates and nitroethane to obtain corresponding β-nitro alcohol products. The results revealed that complex (**35**) is more efficient than (**36**). The yield obtained was 57 and 25%, respectively.

Another reaction catalyzed by CPs is Knoevenagel condensation, which consists of the formation of C–C bonds during condensation of an aldehyde or ketone with active methylene groups under acidic or alkaline conditions [76]. For example, complex [Cd(1,2-bdc-OH)(DMF)_2_·DMF]_n_ (**37**) was tested as heterogeneous catalyst in Knoevenagel condensation to obtain benzylidene malononitrile. The results indicated that small amounts of complex produce, within three minutes, a rapid conversion to 82%, which after 30 min, reaches 92%, and after 60 min, is up to 94%. These findings are more so an indicator of the excellent catalytic activity of (**37**) as it is used in small amounts and it presents tolerable reusability after four cycles [77].

Complex {(H_2_O)_2_[Cd_3_(2,7-cdc)_4_]∙3DMF∙4H_2_O}_n_ (**38**) (2,7-H_2_cdc = 9H-carbazole-2,7-dicarboxylic acid) composed from negatively charged 3D frameworks with 2-fold interpenetration was reported [78] to present catalytic activity in a Knoevenagel reaction, more specific for reactions between benzaldehyde and different methylene substrates in a DMF medium. The results were satisfactory since for malononitrile, the conversion after 2 h reached 100% at room temperature. 

Investigation of the catalytic properties of cadmium (II) CPs lead to the complex {[Cd_3_(bidc)_2_(DMF)_2_(H_2_O)_2_]·H_2_O}_n_ (**39**) (H_2_bidc = 1,3-bisbenzyl-2-imidazolidine-4,5-dicarboxylic acid), which is successfully used for a coupling reaction of benzaldehyde, phenylacetylene, and piperidine with 1,4-dioxane as the solvent [79]. Studies evidenced that the conversion of benzaldehyde under this catalyst activity after four successive cycles was 90.9, 72.6, 52.6, and 46.1% at 120 °C for 12 h.

Karmakar and coworkers [80] reported that two CPs, namely [Cd(paip)(NMF)_2_]_n_ (**40**) (H_2_paip = 5-{pyren-1-ylmethyl)amino}isophtalic acid; NMF = *N*-methylformamide) and {[Cd(aaip)(DMF)(H_2_O)_2_]·H_2_O}_n_ (**41**) (H_2_aaip = 5-{anthracen-9-ylmethyl)amino}isophtalic acid), act as heterogeneous catalysts for the microwave-assisted solvent-free Strecker-type cyanation of acetals in the presence of trimethylsilyl cyanide. The importance of this reaction consists of the incorporation of a cyanide group into a molecule, with the possibility to be further converted into various compounds with extended properties. Comparing the catalytic activity after the same reaction time, (**40**) exhibits a higher activity (yield 95%) than (**41**) (yield 84%). Also, both CPs are recyclable for at least four cycles. 

In addition, complex {[Cd(hipamifba)(H_2_O)_2_∙2H_2_O}_n_ (**42**) (H_2_hipamifba = 4-(((4-((carboxymethyl)carbamoyl)-phenyl)amino)methyl)benzoic acid) (Figure 3c) has proven its efficacy as a heterogeneous catalyst for the Strecker reaction involving aromatic and cyclic ketones and aromatic aldehydes to obtain high yields of α-aminonitriles that are part of various clinical drugs [81]. 

The hydrogen evolution reaction (HER) is the easiest electrochemical manner of producing high-purity hydrogen using a catalyst [82]. The importance of this reaction is easily understood, even more so as hydrogen is an important fuel for the future. Also, finding proper catalysts is an important matter. Therefore, CP {[Cd_2_(ddb)(Hbimb)]∙3H_2_O}_n_ (**21**) (Hddb = 3,5-di(2′,4′-dicarboxylphenyl)benzoic acid; Hbimb = *ortho*-bis(imidazole-1-ylmethyl)benzene) has been tested for this and the results supported its potential as an electrocatalyst for HER [63]. 

On top of the catalytic effect of CPs over different types of chemical processes (Henry reaction, Knoevenagel condensation, cyanosilylation, etc.), there is also their catalytic performances on the degradation of different organic dyes. The challenge with organic dyes is to find the suitable catalyst for their decomposition since they are broadly used in various domains due to accessible costs and the variety of colors, but are hazardous for the environment (they degrade slowly and are nontoxic).

For example, Etaiw and coworkers [83] reported that complex {[Cd_3_(pyzdca)_6_(H_2_O)_4_]·8H_2_O}_n_ (**43**) (Hpyzca = pyrazine-2-carboxylic acid) functions as a heterogeneous catalyst for the degradation of acid blue dye (AB-92) in the presence of H_2_O_2_ as an oxidant. The degradation efficiency of the dye studied is 80% after 240 min; meanwhile, complete degradation occurs after 300 min.

Furthermore, several authors reported the catalytic activity of CPs for degradation of methylene blue (MB), a dye used in the textile industry and associated with water pollution effects. For instance, Hao et al. [84] reported two CPs that photocatalyze the degradation of MB under UV irradiation and formulated [Cd(dctp)(bix)]_n_ (**44**) (H_2_dctp = 2,5-dichloroterephtalic acid; bix = 1,4-bis(imidazol-1-ylmethyl)benzene) and [Cd(bpdc)(bix)_2_]_n_ (**45**) (H_2_bpdc = biphenyl-4,4′-dicarboxylic acid). The degradation efficacy was 75.1% for (**44**) and 84.3% for (**45**) after 120 min of irradiation under a 300 W mercury lamp. Also, CP [Cd_2_(hfpd)(mbp)_2_]_n_ (**46**) (H_4_hfpd = 4,4′-(hexafluoroisopropylidene)diphtalic acid; mbp = 1,5-bis(2-methylbenzimidazol-1-yl)pentane) was reported due to its effects as a heterogeneous catalyst for MB degradation under UV irradiation [85]. After 180 min, dye degradation in the presence of (**46**) occurred with an efficiency of 94.1%, finally being decomposed to smaller inorganic products. 

The exploration of the photocatalytic effects of CPs was extended to methyl violet (MV) degradation by Cai and coworkers [53], who investigated the degradation of MV in polluted waters under effect of complex (**11**). Also, complexes (**25**) and (**26**) were reported due to their high catalytic activity for MV dye degradation [65].

The photocatalytic performances of {[Cd_2_(1,2-bdc)(bmi)_2_]·4H_2_O·DMF}_n_ (**47**) (1,5-bis(2-methylimidazolil-1-yl)pentane) for the degradation of MV was also explored also by other authors [86]. Studies evidenced that after 40 min of exposure to UV light in the presence of (**47**), 47% of MV was decomposed, while in control experiments, photodecomposition of MV was limited to 11% in the same circumstances.

The photocatalytic decomposition of MV and rhodamine B (RhB) under the influence of complex [Cd(1,2-bdc)(bip)(H_2_O)]_n_ (**48**) (bip = 1,3-bis(2-methyl-imidazol-1-yl)propane) was tested [87]. The results evidenced that the rate of MV photodegradation is slower than the RhB rate. This behavior was allegedly related to the nature of dyes. In addition, the degradation of MV and RhB was not observed, not even after 40 min under natural illumination/dark conditions or UV light without a catalyst. The photocatalytic behavior of the compound after three cycles was similar to that observed for the fresh catalyst. CP {[Zn_2_(pa)_2_(bip)_2_]∙6H_2_O}_n_ presents a lower photocatalytic effect for MV and RhB compared to the behavior of (**48**), evidenced by lower rate constants.

Degradation of methyl orange (MO) in the Fenton-like process with H_2_O_2_ was explored for CPs [Cd_2_(tca)(Htca)_0.5_(bbmb)_2_(H_2_O)]_n_ (**49**) (H_3_tca = tricarballylic acid; bbmb = 4,4′-bis(benzimidazol-1-ylmethyl)biphenyl) and {[Cd_2_(suc)_2_(bbmb)_2_(H_2_O)]·H_2_O}_n_ (**50**) (H_2_suc = succinic acid, bbmb = 4,4′-bis(benzimidazol-1-ylmethyl)biphenyl) (Figure 3d) [88]. The catalytic effect consists of the formation of hydroxyl radicals, which present potential for organic pollutant degradation. The degradation efficiency of MO under the influence of (**49**) and (**50**) increased along with the reaction time to 92.8% and 85.4%, respectively. In the absence of a catalyst, self-degradation of MO reached 16.6%.

Furthermore, decomposition of MO in the Fenton-like process with persulfate anions was also catalyzed by the complex [Cd_2_(4-cpa)_4_(bip)_2_]_n_ (**51**) (Hcpa = 4-chlorophenylacetic acid; bip = 1,3-bis(2-methyl-imidazol-1-yl)propane), and total degradation was 60.1% [89].

The Cd(II) CP {[Cd(Hipa)(Hiz)(H_2_O)_2_]⋅3H_2_O}_n_ (**52**) (H_3_ipa = 5-hydroxy-isophthalic acid; Hiz = imidazole) was recently obtained under solvothermal conditions. This complex and its ZnO-doped composite material exhibit dual behavior as adsorbents and photocatalysts of methyl blue (MB), methyl orange (MO), and crystal violet (CV) dye degradation. It is worth mentioning that the composite exhibits a slightly improved surface area with a reduced photogenerated electron–hole ratio, hence displaying enhanced photodegradation activity [90].

Valuable heterogeneous catalysts for acetalization, cyanosilylation, Henry or Strecker reactions, and Knoevenagel condensation processes were developed based on good Lewis acidity of Cd(II) centers from CBCPs. Furthermore, such species show the ability to remove a wide range of pollutants dyes (AB-92, MB, MV, RhB, and MO) from wastewater through photocatalysis assisted or not by oxidants such hydrogen peroxide or persulfate by Fenton-like processes. The Cd-CBCPs discussed significantly increased the degradation efficiency of organic dyes to over 90% in many cases.

The species with catalytic activity are neutral complexes that exhibit good stability in water within a wide pH range to provide several recycling runs. Moreover, these are CP or MOF microporous materials with structural features that ensure a good accessibility of precursors to Cd(II) centers. The differences come from the shape and the dimension of the pore, which as a result, accommodate well to the shape and size of a certain substrate.

The catalytic mechanism is based on the coordination of one or both species involved in the process at Cd(II) Lewis acid centers and their further activation. Instead of photocatalysis, the mechanism consists of electron excitation from HOMO to LUMO of Cd-CBCPs, under UV irradiation. In the next step, the generated electrons are transferred to the surface of the material where they produce ⋅O_2_^−^ from O_2_. Finally, the superoxide in interaction with water molecules produces the ⋅OH active species that degrade the organic pollutants.

### 2.3. Adsorbent Materials Based on Porous Cadmium (II) Carboxylate Coordination Polymers 

The design of CPs with versatile structural features, in particular with high surface areas and cavities of certain dimensions, has drawn a lot of attention lately, mainly due to their applications. Therefore, such CPs are able to either selectively adsorb gases or to trap organic molecules (dyes, solvents, and drugs) [91].

Considering all these aspects, the literature reports many CPs that are able to adsorb CO_2_. This behavior is very important, since CO_2_ is a major greenhouse gas that contributes to climate changes, thus causing global warming. For example, Agarwal and Mukherjee [92] reported one-dimensional CP-formulated {[Cd(1,3-bdc)(npbi)(H_2_O)]∙2H_2_O}_n_ (**53**) (npbi = 1,1′-(4-nitro-1,3-phenylene)bis(1H-benzo[d]imidazole), which presents the ability to selectively adsorb CO_2_ into 1D channels. To evaluate its adsorption properties, the complex was firstly desolvated by being heated it at 100 °C. Then, it was tested for this using several gases (CO_2_, CH_4_, Ar, N_2_, H_2_) in various conditions. The best results were obtained with CO_2_ adsorption and they indicated that volumetric adsorptions of this greenhouse gas by activated CP (**53**) are 80, 30, and 23 mL g^−1^ at 195, 273, and 298 K, respectively. A similar Zn(II) CP, {[Zn(1,3-bdc)(npbi)]·H_2_O}_n_, was not suitable for gas adsorption because of its smaller 1D channels.

**Figure 4:**
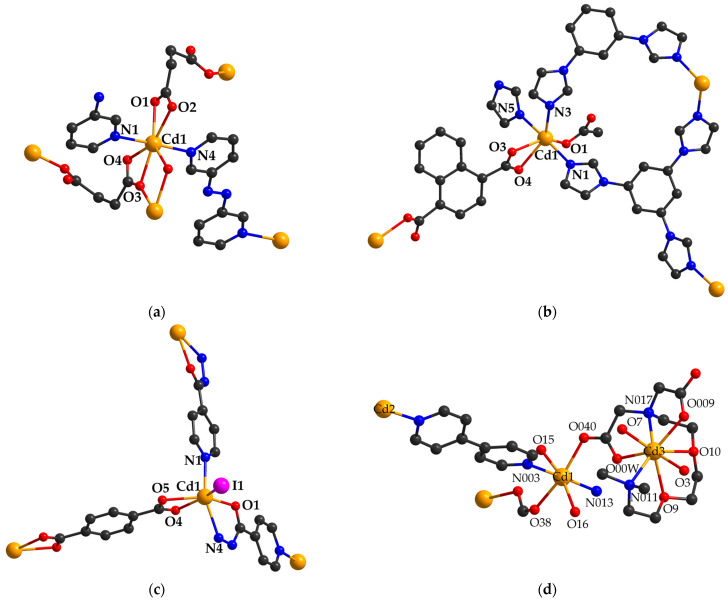
The coordinative site of complexes (**54**) (**a**), (**59**) (**b**), (**62**) (**c**), and (**69**) (**d**) (adapted from refs. [93,96,99,105]).

Haque and co-workers [93] investigated the adsorptive properties for CO_2_ and N_2_ of several CPs; 3,3-azobispyridene is found in their composition, which has free azo functional groups inside the pores that could present interactions with gases. So, CPs {[Cd(suc)(3,3′-azbpy)]∙(MeOH)}_n_ (**54**) (H_2_suc = succinic acid; 3,3′-azbpy = 3,3-azobispyridene) (Figure 4a), [Cd(msuc)(3,3′-azbpy)]_n_ (**55**) (H_2_msuc = methyl succinic acid), and {[Cd(2,2′-dmg)(3,3′-azbpy)(H_2_O)]∙2H_2_O}_n_ (**56**), after desolvation by thermal activation, do not retain N_2_. The authors in [84] suggested that this behavior could be either a consequence of the smaller size in comparison with the diameter of the N_2_ molecule, or of the lack of attraction. As for the CO_2_ adsorption, the most efficient (27.9 cm^3^ g^−1^) CP was found to be (**54**). Complexes (**55**) and (**56**) presented lower adsorption properties for CO_2_ than (**54**) that were explained by the lack of any guest solvent molecules and the two-fold interpenetrated structure, respectively. 

The CP {[Cd(1,4-ndc)_0.5_(pca)]_n_ (**57**) (Hpca = 4-pyridincarboxylic acid; 1,4-H_2_ndc = 1,4-naphtalenedicarboxylic acid) presents a 3D porous framework with large voids (34.9% per unit cell volume) occupied by solvent molecules. After desolvation, it has been found that (**52**) exhibits a high CO_2_ selective uptake in comparison with H_2_, O_2_, Ar, N_2_, and CH_4_ [94]. Preferential CO_2_ sorption in comparison with N_2_ has also been evidenced for {[Cd(Hbtc)(bpp)]∙1.5DMF∙2H_2_O}_n_ (**58**) (bpp = 1,3-bis(4-pyridyl)propane) [95].

In addition, complex {[Cd(1,4-ndc)(tib)]∙3H_2_O}_n_ (**59**) (Figure 4b) also retains CO_2_ at 298 K. The challenge here is posed by the conversion of CP into a variant able to function at room temperature [96].

Besides CO_2_ adsorption, CPs may retain N_2_, H_2_, or CH_4_. For instance, porous three-dimensional CP {[Cd(bpydb)]·6H_2_O}_n_ (**60**) (H_2_bpydb = 4,4′-(4,4′-bipyridine-2,6-diyl) dibenzoic acid) has proven its adsorption properties over and above the mentioned gases, after water molecule removal. The pore volume found was 0.27 cm^3^∙g^−1^ [97].

Porous CP formulated as {[Cd_4_(bcpbp)_3_Cl_6_][CdCl_4_]guest}_n_ (**61**) (bcpbp = 1,1′-bis(4-carboxyphenyl)-4,4′-bipyridinium) is not able to adsorb N_2_, but it retains CH_3_-OH, H_2_O, and CO_2_ at 298 K [98]. The evaluation of the accessible pore volume based on the maximum adsorbed quantity of H_2_O and MeOH was 0.10 cm^3^∙g^−1^ and 0.13 cm^3^∙g^−1^, respectively. In addition, (**61**) presents a great adsorption property over NH_3_ of 0.39 g∙g^−1^ (22.3 mmol∙g^−1^) after the first adsorption cycle, and in the following cycles, it is still able to adsorb important amounts of NH_3_ (0.29 g∙g^−1^; 17 mmol∙g^−1^). 

An interesting and useful application similar to CP is the capturing of iodine for use as medicine. Consequently, Naskar and his team [99] reported that complex {[Cd_2_I_2_(1,4-bdc)_2_(inh)_2_]∙2DMF·H_2_O}_n_ (**62**) (inh = isoniazid) (Figure 4c) is able to reversibly uptake iodine from an organic medium (more than 98%). The structural analysis of CP (**62**) showed polycatenation with microporous channels, while the size of the pores was 17.2x8.31Å^2^ and filled with lattice H_2_O and DMF molecules. After removal of the solvent molecules by heating at 120 °C, the resultant species was able to uptake both iodine and N_2_.

A MOF with a rod-packing framework {(Me_2_NH_2_)_3_[Cd_5_(atnc)_6_]·18DMA·2H_2_O}_n_ (H_3_atnc = 1-amino-2,4,6-tris(5-naphtalenecarboxylic) acid; DMA = *N*,*N*-dimethylacetamide) (**63**), built up from a rigid tricarboxylate ligand, represents a promising adsorbent material applied for the separation and purification of C_2_ hydrocarbons. The abundant pore surface, the uncoordinated amine groups, and the partitioned pore space of a suitable size act synergistically to provide both selective recognition ability and the confinement effect towards C_2_ hydrocarbons for this compound. As a result, it displays promising potential for adsorptive separation of C_2_–CH_4_ and C_2_–CO_2_ mixtures. In addition, the MOF skeleton remains intact in aqueous solutions within a wide pH range of 3 to 11 [100].

The series of CPs [Cd(bdc-NO_2_)(bmip)]_n_ (**64**) and [Cd(bdc-Br)(bmip)]_n_ (**65**) (H_2_bdc-NO_2_ = 2-nitro-1,4-benzenedicarboxylic acid, H_2_bdc-Br = 2-brom-1,4-benzenedicarboxylic acid acid, bmip = bis(2-methylimidazolyl)propane) were developed as porous materials with a BET (Brunauer–Emmett–Teller) surface area of 103 and 283 m^2^ g^−1^, respectively. These compounds demonstrate a gate-opening behavior of ethylene adsorption at low pressures and highly selective adsorption of benzene over cyclohexane or lower alcohols [101].

Besides the adsorption properties over different gaseous molecules, the literature presents an extended series of CPs able to adsorb organic dyes. This behavior gained interest more so as organic dyes are water pollutants and pose an environmental risk. In addition, the use of magnetic adsorbents presents a lot of advantages in this matter. Hence, a CP that contains 1,2,3,4-benzenetetracarboxylate formulated as [Cd(btca)(ppz)]_n_ (**66**) (H_4_btca = 1,2,3,4-benzene tetracarboxylic acid, ppz = piperazine) was magnetized with iron oxide nanoparticles and it was subjected to investigation of adsorption behavior of MB and Chicago Sky Blue (CSB) [102]. After assaying dosage, pH, shaking time, equilibrium, and thermodynamic and kinetic studies, it has been evidenced that (**66**) presented more selective removal of CSB in comparison with MB, while the adsorption capacity was of 64 and 3.6 mg g^−1^, respectively.

A CP that resulted from H_2_pda and 4,4′-azobis(pyridine) (abpy), [Cd(1,2-pda)(abpy)_0.5_(H_2_O)]_n_ (**67**) was reported due to its ability to adsorb MB from an aqueous solution [103]. The adsorption capacity of complex (**67**) within 240 min was 315.2 mg/g, which was almost double that found for a similar Zn-containing CP. In addition, after 240 min, the MB adsorbed quantity decreased, and at the end (after 1440 min), it was found as 5.2 mg g^−1^, suggesting that MB was desorbed.

An acetate-based CP, [Cd_4_(CH_3_COO)(μ-OH)_4_(C_2_H_5_OH)]_n_ (**68**), has been employed in adsorption tests of aromatic dyes [104]. It has been found that CP (**68**) presents a maximum adsorption capacity at a basic pH. Thus, adsorption efficacy is 62.7% at pH 11 and 18.5% at pH 3. In addition, the maximum adsorption property was observed at room temperature. The authors stated that hydrogen bonds, electrostatic interactions, ion exchange, and π–π interactions are involved in physical adsorption.

Other CPs constructed with a flexible carboxylate ligand, ethylene bis(oxyethylenenitrilo)tetraacetic acid (H_4_egta), were used to investigate the adsorption of anionic dyes, particularly MO and Congo Red (CR) [105]. The species used for this purpose were formulated as follows: {[Cd_5_(egta)_2_(4,4′-bpy)_4_(H_2_O)_4_](NO_3_)_2·_4,4′-bpy·8H_2_O}_n_ (**69**) (Figure 4d)**,** {[Cd_3_(egta)(1,2-dpe)_1.5_(H_2_O)_3_](NO_3_)_2_·6H_2_O}_n_ (**70**) (1,2-dpe = 1,2-di(4-pyridyl)ethane), and {[Cd_2_(egta)(dpe)(H_2_O)]·4H_2_O}_n_ (**71**) (dpe = 1,2-di(4-pyridyl)ethylene). The evaluation of dye adsorption properties evidenced that (**69**) and (**70**) easily adsorb MO and CR within 30 and 90 min, respectively, in comparison with (**71**), which adsorbs the mentioned dyes in approximatively 24 h. This behavior is related to the cationic nature of (**69**) and (**70**) and greater non-covalent interactions in comparison with (**71**)**.** Also, the ability of CPs (**69**)**,** (**70**), and (**71**) to adsorb cationic dyes is negligible.

Furthermore, CPs formulated as [Cd(amoip)(FA)_2_]_n_ (**72**) (FA = formamide) and [Cd_2_(pmoip)_2_(MeOH)_2_]_n_ (**73**) were obtained by the solvothermal reactions of Cd(NO_3_)_2_·4H_2_O with 5-{(anthracen-9-ylmethyl)amino}isophthalic acid (H_2_amoip) and 5-{(pyren-1-ylmethyl)amino}isophthalic acid (H_2_pmoip), respectively. These novel CPs were employed for the adsorptive removal of various toxic organic dyes like CR, MB, MV, RhB, and rhodamine 6G (Rh6G) in an aqueous medium. Both CPs are highly effective for the removal of cationic dyes, compared to anionic dyes. However, the two-dimensional pyrene group containing (**73**) shows a higher efficiency of 75–97% in removing all cationic dyes [106].

Another interesting behavior of Cd(II) CPs is their ability to remove different drugs by adsorption. For example, complex [Cd_2_(btc)_2_(phen)_2_(H_2_O)_2_]_n_ (**16**) has been used to remove diclofenac from drinking and tap water [58]. Studies have demonstrated its efficiency on this, and the maximum adsorption capacity was 1822 mg g^−1^.

Therefore, Cd(II) CP (**22**) has been tested as an adsorbent for TEC [64]. Based on the large specific surface area (171.72 m^2^ g^−1^) and pore volume (0.31 cm^3^ g^−1^), this compound adsorbed 64.493 mg g^−1^ of TEC at pH 7 and 298 K. In addition, its pore size favor TEC entering and binding to the adsorption site; the authors indicated a π–π interaction between these species. 

Luo and coworkers [107] reported the adsorbent properties of CP [Cd_3_(dpa)(HCO_2_)(bpp)_3_]_n_ (**74**) (H_2_dpa = diphenic acid; bpp = 1,3-bis(4-pyridyl)propane) for dichromate anions. This property was useful for removing Cr(VI) oxyanion from water. The adsorption of dichromate reached an equilibrium after 15 h, and the maximum quantity adsorbed was 2.451 mg g^−1^. 

Some Cd(II)-CBCPs with proper channels and pores are able to perform the selective adsorption of gases (CO_2_, N_2_, CH_4_, and H_2_) or retain oxoanions (Cr_2_O_7_^2−^), organic toxic species like solvents (methanol), dyes (MB, MO, CSB, RhB/6G, and CR), and drugs (diclofenac, tetracycline) from different media. This ability of Cd(II) CPs can be exploited to reduce the greenhouse effect by capturing CO_2_ and CH_4_, to separate a certain gas from a mixture or to depollute wastewater from industry or hospitals. Some MOF species have exhibited their potential to act as adsorbents of C_2_-CH_4_ and C_2_-CO_2_ mixtures.

Most compounds described above as adsorbent materials are neutral species that exhibit a microporous and robust network, and are stable in water and in a wide pH range. Their high porosity coupled with a good surface area make these materials proficient adsorbent for gases or a liquid-phase adsorbate molecule. Their differences come from the shape, pore dimensions, and core charge that, as result, lead to selective uptake related to the size and charge of the retained species. Moreover, the nature of electrostatic or non-covalent interactions (hydrogen bonds and π–π interactions) with uncoordinated groups as well as the ion exchange involved in adsorption are different from one compound to another. It is worth mentioning that for such species, it is also important to preserve their crystalline structure after an adsorption−desorption cycle.

The mechanism of adsorption consists of electrostatic and/or non-covalent interactions between Cd(II)-CBCPs and guest molecules, except for charged dyes where an ion exchange process can be involved. 

### 2.4. Cadmium (II) Carboxylate Coordination Polymers with Miscellaneous Applications

The Cd(II) CPs present comprehensive application domains, as depicted in the sections presented above. However, there are several applications that cannot be presented in a structured manner, and they are therefore gathered and described here.

For example, compound (**51**) has been tested for its herbicidal activity [89]. To accomplish this objective, the inhibitory rate was evaluated in comparison with the ligand over *Brassica napus* L. and *Echinochloa crusgalli* L. at different concentrations. The rates of inhibition of *Brassica napus* L. root growth was 29.4–39.3, 22.8–30.9, and 10.1–14.2% at 100, 50, and 10 ppm, respectively, suggesting low herbicidal activity against this plant. Contrariwise, the inhibition rates of *Echinochloa crusgalli* L. root growth were high at 100 and 50 ppm (90.3–93.5 and 83.1–88.6). The results indicated that CP (**51**) presents a superior inhibitory rate in comparison with the ligand and suggest the possibility to use it as a herbicide.

Furthermore, complex {[Cd(4-cpha)(bpp)_2_(H_2_O)]∙(4-cpha)∙(H_2_O)}_n_ (**75**) (4-Hcpha = 4-chlorophenoxyacetic acid; bpp = 1,3-bis(4-pyridyl)propane) has been screened for phytogrowth-inhibitory activity against *Brassica campestris* L. and *Echinochloa utilis Ohwi et Yabuno* at several concentrations (20, 40, 60 ppm) [108]. The results indicated that the species present strong effects on *B. Campestris* L. and *E. utilis Ohwi et Yabuno*, inhibiting the germination of seeds at 60 ppm. The overall phytogrowth-inhibitory activity of the CP investigated was superior to that of ligand 4-Hcpha. The authors suggested that Cd(II) ions could enhance the ligand activity. 

A CP resulting from a self-assembly reaction between Cd(II) and ligands 5-methoxyphtalate (5-meo-ip) and 1,3-bis(2-methyl-imidazol-1-yl)propane (bip), formulated as [Cd(5-meo-ip)(bip)]_n_ (**76**), has been reported due to its treatment of uterine fibroids combined with ultrasound therapy [109]. In addition, a similar CP with Zn(II) presented much weaker biological activity than (**71**).

**Figure 5:**
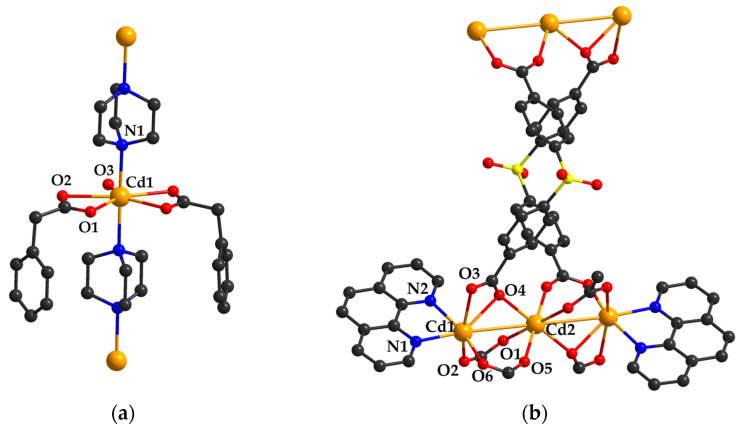
The coordinative site of complexes (**77**) (**a**) and (**78**) (**b**) (adapted from ref. [110,111]).

The bactericidal potential of CP [Cd(phac)_2_(Dabco)(H_2_O)]_n_ (**77**) (Hphac = phenylacetic acid; dabco = 1,4-diazabicyclo[2.2.2]octane) (Figure 5a) has been evidenced against *Staphylococcus aureus* and *Escherichia coli.* Investigation of inhibitory effect of CP components revealed significant inhibitory effect of cadmium salts in comparison with Phac and pillars which exhibit lower effects and absence of activity, respectively [110]. The antibacterial activity was evaluated as diameter of the inhibition zone and evidenced a slightly better activity on *S. aureus* strains. 

Besides researches on the above-mentioned properties, there are also researches related to dielectric properties of CPs and their potential use as gates dielectric in electronic devices. Thus, complexes {[Cd_3_(sba)_2_(phen)_2_(CH_3_COO)_2_]∙2DMA}_n_ (**78**) (H_2_sba = 4,4′-sulfonyldibenzoic acid) (Figure 5b) and {[Cd(fba)(phen)]∙DMA}_n_ (**79**) (H_2_fba = 4,4′-(hexafluoroisopropylidene)bisbenzoic acid) have been reported related with such a behavior. Therefore, CPs present high dielectric constants determined by polar guest molecules in the voids combined with Cd(II)-carboxylate chains. Furthermore, authors [111] stated that dielectric properties are determined by the hydrogen-bond interaction, ordered or disordered solvent molecules.

Lately, to respond to the ever-increasing need for new energy sources, researches focused on proton conductive materials, in particular to CPs with such properties. Consequently, two CPs formulated {[Cd(cbia)_2_(H_2_O)_4_]∙2H_2_O}_n_ (**80**) (Hcbia = 2-(1-carboxymethyl)-1H-benzo[d]imidazol-3-ium-3-yl)acetate) and {[Cd_2_(cbia)_2_(4,4′-bpy)_2_(H_2_O)_2_]∙(cbia)∙(OH)∙2H_2_O}_n_ (**81**) were reported due to their super high proton conductivities [112]. The key elements for such materials are continuous hydrogen bonded network and ligands with hydrophilic units and from this perspective (**80**) and (**81**) contain HCBIA ligand that fulfill the requirements to generate proton-conductive CPs. At 100 °C and 98% relative humidity (RH), proton conductivities of (**80**) and (**81**) are 5.09 × 10^−3^ and 3.41 × 10^−3^ S∙cm^−1^, respectively.

Considering the porous nature of complex [Cd(3-pbi)(DMF)]_n_ (**82**) (3-H_2_pbi = 5-(3-pyridin-3-yl)benzamido)isophtalic acid) it has been tested after treatment with selenium powder as cathode host for Li-Se batteries [113]. The resulted electrode presents an initial capacity of 514 mA h g^−1^, and a reversible capacity of 200 mA h g^−1^ at 1C (1C = 675 mA g^−1^) after 500 cycles.

The reaction of cadmium (II) nitrate with anthracene-9-carboxylic acid (Hatc) and 4′-(4-pyridyl)-2,2′:6′,2″-terpyridine (ptpy) led to the generation of [Cd_2_(ptpy)_2.5_(atc)_4_]_n_ (**83**). This 1D polymer was utilized as a precursor for the fabrication of an organic light-emitting diode (OLED) whose emission wavelengths were investigated at different voltages [114].

Besides the catalytic degradation or adsorption by different materials, organic dyes could be removed by flocculation, an efficient route to treat wastewaters. The exploration of new flocculants has led to CP {[Cd(CH_3_COO)_2_(pip)_2_]∙H_2_O}_n_ (**84**) (pip = 2-phenylimidazo[4,5-*f*][1,10]phenantroline) which present high specific and efficient flocculation effect on Congo Red (CR) [115].

Another reported application of Cd(II) CPs is the generation of CdO nanoparticles in certain conditions, species used in optoelectronic applications, solar cells, photodiodes or as gas sensors [116]. Consequently, 3D supramolecular complex [Cd(4-pca)_2_(H_2_O)_4_]_n_ (**85**) was reported [117] as precursor for CdO nanoparticles by employing different routes, such as calcination and decomposition in the presence of oleic acid. The calcination temperature influences the particle size, while low temperatures lead to smaller particles. The addition of oleic acid as a surfactant during calcination generated particles with an average diameter of 70 nm, since only calcination at 500 and 600 °C generated particles with an average diameter of 83 and 92 nm, respectively.

Ramazani and coworkers [118] presented the sonochemical synthesis of CP {[Cd_3_(3-pyc)_4_(N_3_)_2_(H_2_O)]_n_ (**86**) (3-Hpyc = 3-pyridincarboxylic acid) which was used as raw material for CdO nanoparticles synthesis under different conditions. The results indicated that nano-sized CP precursor produces smaller particles of CdO. The average diameter of CdO particles obtained from direct calcination at 500 °C is 77 nm.

**Table 1:** Examples of Cd(II)-carboxylate-based coordination polymers with diverse applications.

Complex	Carboxylate Linker (Bridge Type)/Ancillary Ligand	Synthesis Method/ Characteristics	Application	Ref.
**Species developed as sensors**				
[Cd(bpmta)_0.5_(1,2-bdc)(H_2_O)]_n_ (**1**)	1,2-benzenedicarboxylate (μ_2_)/*N*,*N*′-bis(pyridin-3-ylmethyl)-terephthalamide)	Hydrothermal/EW ^a^ 335 nm	Fe(III), CrO_4_^2−^, Cr_2_O_7_^2−^ and 2,6-DC-4-NA detection in mM limits	[45]
[Cd_3_(bpy)_3_(cia)_2_]_n_ (**2**)	5-((4-carboxybenzyl) oxy) isophthalate (μ_3_)/2,2′-bipyridine	Hydrothermal/EW 308 nm	NBZ detection in μM limits	[46]
{[Cd(H_4_pbitb)]⋅2DMF⋅8H_2_O}_n_ (**3**)	4,4′,4″,4‴-(1,4-phenylenebis (1H-imidazole-2,4,5- triyl))tetrabenzoic acid (μ_4_)	Solvothermal/EW 361 nm	Fe(III), MnO_4_^−^ and TNP detection in μM limits	[47]
{[Cd_2_(1,3-bdc)_2_(H_2_O)_4_(hdn)]_2_H_2_O}_n_ (**4**)	1,3-benzenedicarboxylate (μ_2_)/*N*,*N′*-(hexane-1,6-diyl)dinicotinamide	ydrothermal/EW 400 nm	NBZ detection in mM limits	[48]
{[Cd(mbdc)(hdn)]H_2_O}_n_ (**5**)	5-methy-1,3-benzenedicarboxylate (μ_2_)/*N*,*N*′-(hexane-1,6-diyl)dinicotinamide	hHydrothermal/EW 400 nm	NBZ detection in mM limits	[48]
[Cd(bzimip)(DMF)]_n_ (**6**)	5-(benzimidazole-1-yl)isophthalic acid (μ_2_)/DMF	Solvothermal	STZ, NFZ, and SDZ detection in μM limits	[49]
[Cd_4_(Hbdcpb)_2_(H_2_O)_5_]_n_ (**7**)	2,3-bis(3,5-dicarboxylphenxoy)benzoate (μ_7_)/water	Solvothermal	SDZ detection in μM limits	[50]
{[Cd_2_(Hbdcpb)(2,2′-bpy)_2_(H_2_O)]·6H_2_O}_n_ (**8**)	2,3-bis(3,5-dicarboxylphenxoy)benzoate (μ_5_)/2,2′-bipyridine	Hydrothermal	NFT detection	[50]
[Cd_4_(2,2′-bpy)_4_(Hddpb)_2_·H_2_O]_n_ (**9**)	2,5ʹ-di(3,5-dicarboylphenoxy)benzoic acid (μ_6_)/2,2′-bipyridine	Hydrothermal/EW 330, 390 nm	NFZ detection in μM limits	[51]
{[Cd_2_(btdb)_2_(4,4-bpy)]·DMF}*_n_* (**10**)	4,4′-(benzo[*c*][1,2,5]thiadiazole-4,7-diyl)dibenzoic acid (μ_2_)/4,4′-bipyridine	Solvothermal	L-His	[52]
[Cd(cphi)(Hbpz)]_n_ (**11**)	5-(4-carboxyphenoxy)isophtalate (μ_2_)/3,3′5,5′-tetramethyl-4,4′-bipyrazole	Solvothermal/EW 300 nm	Fe(III) detection in μM limits	[53]
[Cd_2_(dtta)_2_]_n_ (**12**)	2,5-di(1H-1,2,4-triazol-1-yl)terephthalate (μ_6_)	Solvothermal/EW 300 nm	Cu(II) detection in mM limits	[54]
[Cd(3,3′-dmg)(dpam)]_n_ (**13**)	Dimethyl glutarate (μ_2_)/4,4′-dipyridylamine	Hydrothermal/EW 300, 390 nm	NBZ detection in mM limits	[55]
[Cd(4-tkpvb)(5-*tert*-bipa)]_n_ (**14**)	5-tert-butylisophthalate (μ_2_)/1,2,4,5-tetrakis(4-pyridylvinyl)benzene	Solvothermal/EW 380 nm	Hg(II), CrO_4_^2−^, and Cr_2_O_7_^2−^ detection in Ag(I); Al(III) and Cr(III) detection in μM limits	[56]
[Cd(dpttz)(oba)]_n_ (**15**)	4,4′-oxybis(benzoate) (μ_3_)/2,5-di(pyridine-4-yl)thiazolo [5,4-*d*]thiazole	Solvothermal/EW 380 nm	4-NA and CrO_4_^2−^ detection in Ag(I); Al(III) and Cr(III) detection in μM limits	[57]
[Cd_3_(btc)_2_(phen)_3_(H_2_O)_2_]_n_ (**16**)	1,3,5-benzentricarboxylate (μ_2_)/1,10-phenantroline, water	Hydrothermal/EW 425 nm	Cu(II) and Ni(II) ion detection in Ag(I), Al(III) and Cr(III) detection in μM limits without interferences	[58]
[Cd_3_(cpota)_2_(phen)_3_]_n_·5nH_2_O (**17**)	2-(4-carboxyphenoxy)terephthalate (μ_5_)/1,10-phenanthroline	Hydrothermal/EW 290 nm	volatile organic ketones and (CrO_4_^2−^/Cr_2_O_7_^2−^) detection in Ag(I); Al(III) and Cr(III) detection in μM limits	[59]
{[Cd_2_(edda)(phen)_2_]∙H_2_O}_n_ (**18**)	5,5′(ethane-1,2-diylbis(oxy)) diisophthalate (μ_6_)/1,10-phenanthroline	Hydrothermal/EW 310 nm	MnO_4_^−^ and Cr(VI) ions in μM, AA in nM, and Hacac in μM limits	[60]
[Cd_3_(dcpb)_2_(datrz)(H_2_O)_3_]_n_ (**19**)	4-(2′,3′-dicarboxylphenoxy)benzoate (μ_5_ + μ_6_)/3,5-diamino-1,2,4-triazole	Hydrothermal/EW 390 nm	ClO^−^ and Hacac detection in μM limits	[61]
[Cd(bibt)(3,4-tdc)]_n_ (**20**)	3,4-thiophenedicarboxylate/4,7-bi(1*H*-imidazol-1-yl)benzo-[2,1,3]thiadiazole	Solvothermal	SA detection in μM limits	[62]
{[Cd_2_(ddb)(Hbimb)]∙3H_2_O}_n_ (**21**)	3,5-di(2′,4′-dicarboxylphenyl)benzoate (μ_2_ + μ_7_)/*ortho*-bis(imidazole-1-ylmethyl)benzene	Solvothermal	NF selective detection in μM limits	[63]
{[Cd(1,2-pda)(tib)]·H_2_O}_n_ (**22**)	1,2-phenylenediacetate (μ_2_)/1,3,5-tris(1-imidazolyl)benzene	Hydrothermal/EW 280 nm, BET ^b^ 171.72 m^2^ g^−1^	TEC detection in μM limits	[64]
{[Cd_4_(1,3-pda)_4_(tib)_2_(dib)_2_]·7H_2_O}_n_ (**23**)	1,3-phenylenediacetate (μ_2_)/1,3,5-tris(1-imidazolyl)benzene; 1,3-di(1-imidazolyl)benzene	Hydrothermal/EW 333 nm	TEC detection in μM limits	[64]
{[Cd(1,4-pda)(tib)]·4H_2_O}_n_ (**24**)	1,4-phenylenediacetate (μ_2_)/1,3,5-tris(1-imidazolyl)benzene	Hydrothermal/EW 333 nm	TEC detection in μM limits	[64]
[Cd(tdc)(tbb)]_n_ (**25**)	2,5-thiophenedicarboxylate (μ_2_)/1,4-bis(thiabendazole-1-yl)-2-butene	Hydrothermal/EW 345 nm	Fe(III), Hacac, and NOR detection in μM limits	[65]
{[Cd(tpca)(tbb)]·H_2_O}_n_ (**26**)	2,3,5,6-tetrabromoterephtalate (μ_2_)/1,4-bis(thiabendazole-1-yl)-2-butene	Hydrothermal/EW 345 nm	Fe(III), Hacac, and NOR detection in μM limits	[65]
[Cd_3_(bptc)_2_(H_2_O)_4_]_n_ (**27**)	Biphenyl-2,4′,5-tricarboxylate (μ_2_)/water	hydrothermal	Fe(III) and acetone detection	[66]
{[Cd(dint)(1,4-bdc)(H_2_O)]·ACN}_n_ (**28**)	1,4-benzenedicarboxylate (μ_2_)/1,4-di(imidazol-1-yl)naphthalene	Solvothermal/EW of 230 and 290 nm	Vitamin B2 detection in mM limits, nitenpyram and imidacloprid detection in μM limits	[67]
[Cd(pddb)H_2_O]_n_ (**29**)	4,4′-(pyridine-2,6-diyl)-dibenzoic acid (μ_4_)/water	Hydrothermal/EW 340 nm	MEAA detection in μM limits	[68]
{Cd_3_(btc)_2_(btd-bpy)_2_]·1.5MeOH·4H_2_O}_n_ (**30**)	Benzene-1,3,5-tricarboxylate (μ_5_)/bis(pyridin-4-yl)benzothiadiazole	Hydrothermal/EW 320, 335 nm	Ag(I), Al(III) and Cr(III) detection in μM limits	[69]
[Cd_2_(1,4-ndc)_2_(btd-bpy)_2_]n (**31**)	Naphthalene-1,4-dicarboxylate (μ_3_)/bis(pyridin-4-yl)benzothiadiazole	Solvothermal/EW 335, 365 nm	Ag(I), Al(III) and Cr(III) detection in μM limits	[69]
**Species developed as catalysts**				
[Cd(ipc)(Cl)(H_2_O)]_n_ (**32**)	5-imidazol-1-yl-2-pyridine carboxylate (μ_2_)/chloride, water	Hydrothermal/four RR ^c^	Catalyst for acetalization reaction	[71]
{[Cd(cbdcp)(H_2_O)_4_]∙(H_2_O)}_n_ (**33**)	1-(3,5-dicarboxybenzyl)-4,4′-bipyridinium ion (μ_2_)/water	Hydrothermal/five RR	Catalyst for cyanosilylation reaction	[72]
[Cd_2_(1,4-ndc)_2_(DMF)_2_]_n_ (**34**)	1,4-naphtalenedicarboxylate (μ_4_)/*N*,*N*′-dimethylformamide	Solvothermal	Catalyst for cyanosilylation reaction of aromatic aldehydes	[73]
[Cd(3,3′-dbdc)(2,2′-dipy)(H_2_O)]_n_ (**35**)	3,3′-dihydroxy-(1,1′-biphenyl)-4,4′-dicarboxylate (μ_2_)/2,2′-dipyridine	Hydrothermal	Catalyst for Henry reaction	[74]
[Cd(4,4′-dbdc)(phen)]_n_ (**36**)	4,4′-dihydroxy-(1,1′-biphenyl)-3,3′-dicarboxylate (μ_3_)/1,10-phenantroline	Hydrothermal	Catalyst for Henry reaction	[74]
[Cd(bdc-OH)(DMF)_2_·DMF]_n_ (**37**)	2-hydroxyterephtalate (μ_2_)/*N*,*N*′-dimethylformamide	Solvothermal/four RR	Heterogeneous catalyst for Knoevenagel condensation	[77]
{(H_2_O)_2_[Cd_3_(2,7-cdc)_4_]∙3DMF∙4H_2_O}_n_ (**38**)	9*H*-carbazole-2,7-dicarboxylate (μ_2_ + μ_3_ + μ_4_)	Solvothermal/two RR	Catalyst for Knoevenagel condensation	[78]
{[Cd_3_(bidc)_2_(DMF)_2_(H_2_O)_2_]·H_2_O}_n_ (**39**)	1,3-bisbenzyl-2-imidazolidine-4,5-dicarboxylate (μ_3_)/*N*,*N*′-dimethylformamide, water	One pot at heating	Catalyst for aldehyde coupling reaction	[79]
[Cd(paip)(NMF)_2_]_n_ (**40**)	5-{pyren-1-ylmethyl)amino}isophtalate (μ_3_)/*N*-methylformamide	Solvothermal/four RR	Catalysts for solvent-free microwave-assisted cyanation of acetals	[80]
{[Cd(aaip)(DMF)(H_2_O)_2_]·H_2_O}_n_ (**41**)	5-{anthracen-9-ylmethyl)amino}isophtalate (μ_2_)/*N*,*N*′-dimethylformamide	Solvothermal/four RR	atalysts for solvent-free microwave-assisted cyanation of acetals	[80]
{[Cd(hipamifba)(H_2_O)_2_∙2H_2_O}_n_ (**42**)	4-(((4-((carboxymethyl)carbamoyl)-phenyl)amino)methyl)benzoate (μ_2_)/water	One pot at rt by stirring/five RR	hHeterogeneous catalyst for Strecker reaction	[81]
{[Cd_3_(pyzdca)_6_(H_2_O)_4_]·8H_2_O}_n_ (**43**)	Pyrazine-2-carboxylate (μ_2_)/water	One pot at rt by stirring/active in presence of H_2_O_2_	Catalyst for degradation of AB-92	[83]
[Cd(dctp)(bix)]_n_ (**44**)	2,5-dichloroterephtalate (μ_2_)/1,4-bis(imidazol-1-ylmethyl)benzene	Hydrothermal and sonochemical/EW 365 nm	Photocatalytic activity for degradation of MB under UV irradiation	[84]
[Cd(bpdc)(bix)_2_]_n_ (**45**)	Biphenyl-4,4′-dicarboxylate (μ_2_)/1,4-bis(imidazol-1-ylmethyl)benzene	Hydrothermal and sonochemical/UV irradiation	Photocatalytic activity for degradation of MB under UV irradiation	[84]
[Cd_2_(hfpd)(mbp)_2_]_n_ (**46**)	4,4′-(hexafluoroisopropylidene)diphtalate (μ_4_)/1,5-bis(2-methylbenzimidazol-1-yl)pentane	Hydrothermal and sonochemical/EW 365 nm	Photocatalytic activity for degradation of MB under UV irradiation	[85]
{[Cd_2_(btc)(bmi)_2_]·4H_2_O·DMF}_n_ (**47**)	1,2,4,5-benzen-tetracarboxylate (μ_4_)/1,5-bis(2-Methylimidazolil-1-yl)pentane, water, *N*,*N*′-dimethylformamide	Solvothermal/EW 365 nm	Photocatalyst for MV degradation	[86]
[Cd(1,2-bdc)(bip)(H_2_O)]_n_ (**48**)	1,2-benzen-dicarboxylate (μ_2_)/1,3-bis(2-methyl-imidazol-1-yl)propane, water	Solvothermal/EW 365 nm	Photocatalyst for degradation of MV and RhB	[87]
[Cd_2_(tca)(htca)_0.5_(bbmb)_2_(H_2_O)]_n_ (**49**)	Tricarballylic acid anion (μ_2+_μ_3_)/4,4′-bis(benzimidazol-1-ylmethyl)biphenyl	Hydrothermal/active in presence of H_2_O_2_	Catalyst for degradation of MO in Fenton-like process	[88]
{[Cd_2_(suc)_2_(bbmb)_2_(H_2_O)]∙H_2_O}_n_ (**50**)	Succinate (μ_2_)/4,4′-bis(benzimidazol-1-ylmethyl)biphenyl, water	Hydrothermal/active in presence of H_2_O_2_	Catalyst for degradation of MO in Fenton-like process	[88]
[Cd_2_(4-cpa)_4_(bip)_2_]_n_ (**51**)	4-chlorophenylacetate/1,3-bis(2-methyl-imidazol-1-yl)propane	Hydrothermal/active in presence of Na_2_S_2_O_8_	Catalyst for degradation of MO in Fenton-like process	[89]
{[Cd(Hipa)(Hiz)(H_2_O)_2_]⋅3H_2_O}_n_ (**52**)	5-hydroxy isophthalic acid (μ_2_)/imidazole	Solvothermal	Photocatalysts in MB, MO, and CV degradation	[90]
**Species developed as adsorbent materials**				
{[Cd(1,3-bdc)(bc)(H_2_O)]∙2H_2_O}_n_ (**53**)	1,3-benzendicarboxylate (μ_2_)/1,1′-(4-nitro-1,3-phenylene)bis(1*H*-benzo[*d*]imidazole, water	Solvothermal/type I gas sorption isotherm	Adsorption of CO_2_	[92]
{[Cd(suc)(3,3′-azbpy)]∙(MeOH)}_n_ (**54**)	Succinate (μ_3_)/3,3-azobispyridene	Slow diffusion/surface adsorption	Adsorption of CO_2_	[93]
[Cd(msuc)(3,3′-azbpy)]_n_ (**55**)	Methyl succinate (μ_3_)/3,3-azobispyridene	Slow diffusion/surface adsorption	Adsorption of CO_2_	[93]
{[Cd(2,2′-dmglut)(3,3′-azbpy)(H_2_O)]∙2H_2_O}_n_ (**56**)	2,2′-dimethylglutarate (μ_2_)/3,3-azobispyridene, water	Slow diffusion/surface adsorption	Adsorption of CO_2_	[93]
[Cd(ndc)_0.5_(pca)]_n_ (**57**)	2,6-naphtalenedicarboxylate (μ_2_)/4-pyridincarboxylate	Solvothermal/type I gas sorption isotherm	Adsorption of CO_2_	[94]
{[Cd(Hbtc)(bpp)]∙1.5DMF∙2H_2_O}_n_ (**58**)	1,3,5-benzentricarboxylate (μ_2_)/1,3-bis(4-pyridyl)propane	Solvothermal/surface adsorption	Adsorption of CO_2_	[95]
{[Cd(1,4-ndc)(tib)]∙3H_2_O}_n_ (**59**)	1,4-naphtalenedicarboxylate (μ_2_)/1,3,5-tris(1-imidazolyl)benzene	Solvothermal/BET 421.05 m^2^ g^−1^	Adsorption of CO_2_	[96]
{[Cd(bpydb)]·6H_2_O}_n_ (**60**)	4,4′-(4,4′-bipyridine-2,6-diyl) dibenzoate (μ_2_)	Solvothermal/BET 346 m^2^ g^−1^, reversible type I gas sorption isotherm for N_2_ and H_2_	Adsorption of N_2_, H_2_, and CH_4_	[97]
{[Cd_4_(bcpbp)_3_Cl_6_][CdCl_4_]}_n_ (**61**)	1,1′-bis(4-carboxyphenyl)-4,4′-bipyridinium (μ_2_)/chloride	Solvothermal/complex shape of gas sorption isotherms	Adsorption of CO_2_, MeOH, H_2_O, and NH_3_	[98]
{[Cd_2_I_2_(1,4-bdc)_2_(inh)_2_]∙2DMF·H_2_O)}_n_ (**62**)	1,4-benzendicarboxylate (μ_2_)/isoniazid, iodine	Slow diffusion/BET 611 m^2^ g^−1^	Adsorption of I_2_, and N_2_	[99]
{(Me_2_NH_2_)_3_[Cd_5_(atnc)_6_]·18DMA·2H_2_O}_n_ (**63**)	1-amino-2,4,6-tris(5-naphtalenecarboxylate) (μ_6_ + μ_7_)	Hydrothermal/BET 1193 m^2^ g^−1^, type I gas sorbtion isoterm	Adsorption of C2/C1 hydrocarbons and C2/CO_2_ separation	[100]
{[Cd(bdc-NO_2_)(bmip)]·3DMF}_n_ (**64**)	2-nitro-1,4-benzenedicarboxylic acid (μ_2_)/bis(2-methylimidazolyl)propane	Solvothermal/BET of 103 m^2^·g^−1^	Adsorption of ethylene, benzene, cyclohexane, lower alcohols (methanol, ethanol, isopropyl alcohol)	[101]
{[Cd(bdc-Br)(bmip)]·3DMF}_n_ (**65**)	2-brom-1,4-benzenedicarboxylic acid (μ_2_)/bis(2-methylimidazolyl)propane	Solvothermal/BET of 283 m^2^ g^−1^	Adsorption of ethylene, benzene, cyclohexane, lower alcohols (methanol, ethanol, isopropyl alcohol)	[101]
[Cd(btca)(ppz)]_n_ (**66**)	1,2,3,4-benzentetracarboxylate (μ_4_)/piperazine	One pot at rt by stirring/BET of 5.45 m^2^ g^−1^	Adsorption of CSB and MB dyes	[102]
[Cd(pda)(abpy)_0.5_(H_2_O)]_n_ (**67**)	1,2-phenylenediacetate (μ_4_)/4,4′-azobis(pyridine), water	Hydrothermal	adsorption of MB	[103]
{[Cd_4_(CH_3_COO)(μ-OH)_4_]·C_2_H_5_OH}_n_ (**68**)	Acetate (μ_2_)/hydroxide	Solvothermal/type I gas sorption isotherm	Adsorption of MB and MO dyes	[104]
{[Cd_5_(egta)_2_(4,4′-bipy)_4_(H_2_O)_4_](NO_3_)_2·_4,4′-bpy·8H_2_O}_n_ (**69**)	Ethylene bis(oxyethylenenitrilo) tetracetate (μ_2_)/4,4′-bipyridine, water	Solvothermal/	Adsorption of MO and CR dyes	[105]
{[Cd_3_(egta)(1,2-dpe)_1.5_(H_2_O)_3_](NO_3_)_2_·6H_2_O}_n_ (**70**)	Ethylene bis(oxyethylenenitrilo) tetracetate (μ_2_ + μ_4_)/1,2-di(4-pyridyl)ethane, water	Solvothermal/	Adsorption of MO and CR dyes	[105]
{[Cd_2_(egta)(dpe)(H_2_O)]·4H_2_O}_n_ (**71**)	Ethylene bis(oxyethylenenitrilo) tetracetate (μ_2_)/dpe = 1,2-di(4-pyridyl)ethylene, water	Solvothermal/	Adsorption of MO and CR dyes	[105]
[Cd(amoip)(FA)_2_]_n_ (**72**)	5-{(anthracen-9-ylmethyl)amino}isophthalic acid (μ_3_)/formamide	Hydrothermal/three RR, Langmuir isotherm	Adsorption of CR, MB, MV, RhB, and Rh6G	[106]
[Cd_2_(pmoip)_2_(MeOH)_2_]_n_ (**73**)	5-{(pyren-1-ylmethyl)amino}isophthalic acid (μ_4_)/methanol	Hydrothermal/three RR, Langmuir isotherm	Adsorption of CR, MB, MV, RhB, and Rh6G	[106]
[Cd_3_(dpa)(HCO_2_)(bpp)_3_]_n_ (**74**)	Diphenic acid dianion (μ_4_)/formate, 1,3-bis(4-pyridyl)propane	Hydrothermal/Langmuir isotherm	Adsorbtion of Cr_2_O_7_^2−^ anion	[107]
**Species with miscellaneous applications**				
{[Cd(4-cpha)(bpp)_2_(H_2_O)]∙(4-cpha)∙(H_2_O)}_n_ (**75**)	4-chlorophenoxyacetate (μ_2_)/1,3-bis(4-pyridyl)propane, water	Hydrothermal	Phytogrowth-inhibitory activity against *Brassica campestris* L., *Echinochloa utilis Ohwi et Yabuno*	[108]
[Cd(5-meo-ip)(bip)]_n_ (**76**)	5-methoxyisophtalate (μ_3_)/1,3-bis(2-methyl-imidazol-1-yl)propane	Hydrothermal	Uterine fibroid treatment	[109]
[Cd(phac)_2_(dabco)(H_2_O)]_n_ (**77**)	Phenylacetate (μ_2_)/1,4-diazabicyclo [2.2.2]octane	Solvothermal	Antibacterial activity against *S. aureus* and *E. coli*	[110]
{[Cd_3_(sba)_2_(phen)_2_(CH_3_COO)_2_]∙2DMA}_n_ (**78**)	4,4′-sulfonyldibenzoate (μ_4_)/1,10-phenantroline, acetate	Solvothermal/dielectric constant 20.0 (1 kHz)	Gate dielectrics in electronic devices	[111]
{[Cd(fba)(phen)]∙DMA}_n_ (79)	4,4′-(hexafluoroisopropylidene)bisbenzoate (μ_3_)/1,10-phenantroline	Solvothermal/dielectric constant 32.0 (1 kHz)	Gate dielectrics in electronic devices	[111]
{[Cd(cbia)_2_(H_2_O)_4_]∙2H_2_O}_n_ (**80**)	2-(1-carboxymethyl)-1H-benzo[d]imidazol-3-ium-3-yl)acetate (μ_3_)/water	Solvothermal/proton conductivity 5.09 × 10^−3^ S cm^−1^	Material with high proton conductivity	[112]
{[Cd_2_(cbia)_2_(4,4′-bipy)_2_(H_2_O)_2_]∙(cbia)(OH)∙2H_2_O}_n_ (**81**)	2-(1-carboxymethyl)-1H-benzo[d]imidazol-3-ium-3-yl)acetate (μ_2_)/4,4′-bipyridine, water	Solvothermal/proton conductivity 3.41 × 10^−3^ S cm^−1^	Material with high proton conductivity	[112]
[Cd(3-pbi)(DMF)]_n_ (**82**)	5-(3-pyridin-3-yl)benzamido)isophtalate (μ_2_)/*N*,*N*′-dimethylformamide	Solvothermal	hHost for lithium-selenium batteries	[113]
[Cd_2_(ptpy)_2.5_(atc)_4_]_n_ (**83**)	Anthracene-9-carboxylic acid/4′-(4-pyridyl)-2,2′:6′,2″-terpyridine (ptpy)	Sonochemical	OLED fabrications	[114]
{[Cd(CH_3_COO)_2_(pip)_2_]∙H_2_O}_n_ (**84**)	Acetate (μ_2_)/2-phenylimidazo [4,5-*f*][1,10]phenantroline	Solvothermal	loculant for CR	[115]
[Cd(4-pyc)_2_(H_2_O)_4_]_n_ (**85**)	4-pyridincarboxylate anion (μ_2_)/water	Hydrothermal/average particle diameter 77 nm	Raw material for CdO nanoparticles	[117]
{[Cd_3_(3-pyc)_4_(N_3_)_2_(H_2_O)]_n_ (**86**)	Pyridine-3-carboxylate (μ_2_)/azido, water	Sonochemical/average particle diameter 780 nm	Raw material for CdO nanoparticles	[118]

^a^ EW = excitation wavelength; ^b^ BET = specific surface area calculated using Brunauer–Emmett–Teller method; ^c^ RR = recycling runs.

Besides the applications of Cd(II)-CBCPs structured in previous subchapters Section 2.1, Section 2.2 and Section 2.3 and presented extensively in, a several miscellaneous ones have been reported: the herbicidal activity over *Echinochloa crusgalli* L., the phyto-growth inhibitory effects on *B. Campestris* L. and *E. utilis Ohwi et Yabuno*, the bactericidal activities against *S. aureus* and *E. coli*. Also, the potential use for treatment of uterine fibroids associated with ultrasound therapy has been demonstrated for one species. 

Furthermore, finding new energy sources is connected with Cd(II)-CBCPs which may be incorporated into energy storage or electronic devices, or may be converted into CdO nanoparticles suitable for solar cells, or even gas sensors. Studies evidenced that a proper selection of both carboxylate and ancillary ligands, as well as the reaction condition provide Cd CPs with structural features (dielectric behavior, proton conductivity) useful for electronic device design. 

Taking into account all the information related to Cd(II)-CBCPs and presented in this paper, it may be concluded that although Cd(II) ions are not suitable for biological applications due to their intrinsic toxicity, there are many other domains where they have demonstrated their complete usefulness.

## 3. Conclusions

The aim of this paper was to gather and organize the most relevant data regarding Cd(II)-CBCPs, emphasizing the application areas of these species. 

Recent progress in both coordination chemistry and crystal engineering has allowed the design and synthesis of a wide range of Cd(II)-CBCPs with desired structures and properties by choosing a suitable mixture of organic ligands. During recent years, the combination of rigid-backbone polycarboxylate and heterocycle N-donor ligands has been confirmed as one of the most useful building strategies to obtain such derivatives. The polycarboxylate ligands usually act as multiple-Cd(II) center connectors depending on the number of carboxylate groups, the molar ratio, both substituents’ nature, and ancillary ligands with a similar aspect mentioned for Zn-CBCPs [11]. Similar to other similar CPs, both structure and morphology are difficult to control since several factors, such as the molar ratio, synthesis method, reaction conditions (solvent, temperature, and pH), stereochemical versatility of Cd(II), and coordinative ability of the carboxylate and ancillary ligand, are involved. 

A great number of Cd(II)-CBCPs present fluorescence responses towards metallic ions (Ag(I), Cu(II), Ni(II), Al(III), Fe(III), Cr(III), Cr(VI)), anionic species (CrO_4_^2−^/Cr_2_O_7_^2−^, MnO_4_^−^), and organic molecules (2,6-DC-4-NA, AA, Hacac, ketones, NBZ, NF, NFT, NOR, SDZ) encountered in many media, including environmental samples.

Due to their Lewis acid character, Cd(II) ions present excellent catalytic properties and consequently, many reported Cd(II)-CBCPs were subjected to tests of heterogeneous catalytic behavior. Their capability to catalyze under certain conditions has been proven, with satisfying and even high yields from acetalization cyanosilylation and Henry, Strecker, and Knoevenagel reactions. In addition, many Cd(II)-CBCPs with suitable pores and channels into their structures have adsorptive properties for gases, organic molecules, or medicines. Besides these, some Cd(II)-CBCPs have proven herbicidal and bactericidal activities and were further used as raw materials for CdO nanoparticle synthesis.

Finally, this paper provides an overview and gives a summary of current knowledge regarding applications for Cd(II)-CBCPs, which are quite comprehensive and comparable with those of Zn-CBCPs, with the exception of the biological field [11]. In addition, it is worth mentioning that in most cases, the Cd(II)-CBCP properties are a consequence of their structures (dimensions of the pores, hydrogen bonds, presence of certain ligands into composition, etc.).

## 4. Further Perspectives

In recent years, the CP domain has been a very active research area that has advanced considerably and is based on either finding new materials with predefined properties or on improving existing ones. With regard to Cd(II)-CBCPs, design of new species is possible using proper ligands, mainly polycarboxylates with a rigid backbone functionalized with substituents with coordinative sites or combining carboxylic derivatives with other polydentate ligands with donors other than nitrogen atoms. The domain of fluorescence can be extended by including in the Cd(II)-CBCP network some known luminophores such as the lanthanide ions, for which several studies on species similar to Zn(II) have proven their capacity to modulate the optical properties of such derivatives so far. 

Considering the properties already revealed and the applications of Cd(II)-CBCPs, future perspectives are related to the extension of catalytic properties over other reactions besides those already mentioned, or to the improvement of the yield of studied species. 

In addition, the design of species able to adsorb many gases, particularly those with a greenhouse effect, could be an enormous achievement, considering their negative effect on the environment.

Even if the literature data regarding Cd(II)-CBCPs with biological properties are very scarce, a barrier that cannot be overcome is gaining new candidates with biological properties considering the known toxicity of Cd(II) ions. 

Also, involvement of Cd(II)-CBCPs in technologies such as networks of electronic devices or finding new energy sources could be further investigated and extended, since the results reported so far are promising. 

Finally, we must conclude that despite all data cited in this paper being from 2014 to the present date, there is still plenty of room for improvement and development in the Cd(II)-CBCP domain. The information in our literature review may support future exploration studies.

## Abbreviations

AA	Ascorbic acid
H_2_aaip	5-{anthracen-9-ylmethyl)amino}isophtalic acid
AB-92	Acid blue 92 dye
abpy	4,4′-azobis(pyridine)
Hacac	Acetylacetone
ACN	Acetonitrile
H_2_amoip	5-{(anthracen-9-ylmethyl)amino}isophthalic acid
Hatc	Anthracene-9-carboxylic acid
3,3′-azbpy	3,3′-azobispyridene
bbi	1,4-bis(imidazol-1-yl)butane
bbmb	4,4′-bis(benzimidazol-1-ylmethyl)biphenyl
bmip	Bis(2-methylimidazolyl)propane
H_2_bdc	Benzendicarboxylic acid
H_2_bdc-Br	2-brom-1,4-benzenedicarboxylic acid acid
H_2_bdc-NO_2_	2-nitro-1,4-benzenedicarboxylic acid
bcpbp	1,1′-bis(4-carboxyphenyl)-4,4′-bipyridinium
H_5_bdcpb	2,3-bis(3,5-dicarboxylphenxoy)benzoic acid
BET	Brunauer–Emmett–Teller
H_2_bidc	1,3-bisbenzyl-2-imidazolidine-4,5-dicarboxylic acid
bibt	4,7-bi(1H-imidazol-1-yl)benzo-[2,1,3]thiadiazole
bimb	*Ortho*-bis(imidazole-1-ylmethyl)benzene
bip	1,3-bis(2-methyl-imidazol-1-yl)propane
bix	1,4-bis(imidazol-1-ylmethyl)benzene
bpp	1,3-bi(4-pyridyl)propane
2,2′-bpy	2,2′-bipyridine
4,4′-bpy	4,4′-bipyridine
bix	1,4-bis(imidazol-1-ylmethyl)benzene
bmi	1,5-bis(2-methylimidazolil-1-yl)pentane
H_2_bpdc	Biphenyl-4,4′-dicarboxylic acid
bpmta	*N*,*N*′-bis(pyridin-3-ylmethyl)-terephthalamide
bpp	1,3-bis(4-pyridyl)propane
H_3_bptc	Biphenyl-2,4′,5-tricarboxylic acid
H_2_bpydb	4,4′-(4,4′-bipyridine-2,6-diyl) dibenzoic acid
Hbpz	3,3′5,5′-tetramethyl-4,4′-bipyrazole
H_3_btc	1,3,5-benzentricarboxylic acid
H_4_btca	1,2,3,4-benzene tetracarboxylic acid
btd-bpy	Bis(pyridin-4-yl)benzothiadiazole
H_2_bzimip	5-(benzimidazole-1-yl)isophthalic acid
H_3_cbdcpBr	1-(3,5-dicarboxybenzyl)-4,4′-bipyridinium bromide
Hcbia	2-(1-carboxymethyl)-1H-benzo[d]imidazol-3-ium-3-yl)acetate
2,7-H_2_cdc	9*H*-carbazole-2,7-dicarboxylic acid
5-H_3_cia	5-((4-carboxybenzyl) oxy) isophthalic acid
4-Hcpa	4-chlorophenylacetic acid
4-Hcpha	4-chlorophenoxyacetic acid
H_3_cphi	5-(4-carboxyphenoxy)isopthalic acid
H_3_cpota	2-(4-carboxyphenoxy)terephthalic
CR	Congo Red
CSB	Chicago Sky Blue
dabco	1,4-diazabicyclo[2.2.2]octane
datrz	3,5-diamino-1,2,4-triazole
H_4_(3,3′-dbdc)	3,3′-dihydroxy-(1,1′-biphenyl)-4,4′-dicarboxylic acid
H_4_(4,4′-dbdc)	4,4′-dihydroxy-(1,1′-biphenyl)-3,3′-dicarboxylic acid
2,6-DC-4-NA	2,6-dichloro-4-nitroaniline
H_3_dcpb	4-(2′,3′-dicarboxylphenoxy) benzoic acid
H_2_dctp	2,5-dichloroterephtalic acid
H_5_ddb	3,5-di(2′,4′-dicarboxylphenyl)benzoic acid
H_5_ddpb	2,5ʹ-di(3,5-dicarboylphenoxy)benzoic acid
dib	1,3-di(1-imidazolyl)benzene
dint	1,4-di(imidazol-1-yl)naphthalene
DMA	*N*,*N′*-dimethylacetamide
DMF	*N*,*N′*-dimethylformamide
H_2_dmg	2,2′-dimethyl glutaric acid
H_2_dpa	Diphenic acid/dibenzoic acid
dpam	4,4′-dipyridylamine
1,2-dpe	1,2-di(4-pyridyl)ethane
dpe	1,2-di(4-pyridyl)ethylene
dpttz	2,5-di(pyridine-4-yl)thiazolo[5,4-*d*]thiazole
H_4_edda	5,5′(ethane-1,2-diylbis(oxy)) diisophthalic acid
EW	Excitation wavelength
H_4_egta	Ethylene bis(oxyethylenenitrilo)tetraacetic acid
FA	Formamide
H_2_fba	4,4′-(hexafluoroisopropylidene)bisbenzoic acid
H_2_glut	Glutaric acid
HER	Hydrogen evolution reaction
hdn	*N*,*N*′-(hexane-1,6-diyl)dinicotinamide
H_4_hfpd	4,4′-(hexafluoroisopropylidene)diphtalic acid
H_2_hipamifba	4-(((4-((carboxymethyl)carbamoyl)-phenyl)amino)methyl)benzoate
HOMO	Highest occupied molecular orbital
inh	Isoniazid
H_3_ipa	5-hydroxy isophthalic acid
Hipc	5-imidazol-1-yl)-2-pyridine carboxylic acid
Hiz	Imidazole
LUMO	Lowest unoccupied molecular orbital
MB	Methylene blue
H_2_mbdc	5-methy-1,3- benzenedicarboxylic acid
mbp	1,5-bis(2-methylbenzimidazol-1-yl)pentane
MeOH	Methanol
5-meo-H_2_ip	5-methoxyisophtalic acid
MO	Methyl orange
H_2_msuc	Methyl succinic acid
MV	Methyl violet
4-NA	4-nitroaniline
NBZ	Nitrobenzene
H_2_ndc	Naphtalenedicarboxylic acid
NF	Nitrofuran
NFT	Nitrofurantoin
NFZ	Nitrofurazone
NMF	N-methylformamide
NOR	Norfloxacin
NPBI	1,1′-(4-nitro-1,3-phenylene)bis(1H-benzo[d]imidazole
H_2_oba	4,4′-oxybis(benzoic acid)
OLED	Organic light-emitting diode
H_2_pda	1,2-phenylenediacetic acid
H_2_pa	Pamoic acid
H_2_paip	5-{pyren-1-ylmethyl)amino}isophtalic acid
3-H_2_pbi	5-(3-pyridin-3-yl)benzamido)isophtalic acid
pc1	1,1′-bis(4-carboxyphenyl)-4,4′-bipyridinium
Hpca	4-pyridincarboxylic acid
Hphac	Phenylacetic acid
phen	1,10-phenantroline
pip	2-phenylimidazo[4,5-*f*][1,10]phenantroline
H_2_pmoip	5-{(pyren-1-ylmethyl)amino}isophthalic acid
Ppz	Piperazine
ptpy	4′-(4-pyridyl)-2,2′:6′,2″-terpyridine
3-Hpyc	3-pyridincarboxylic acid
Hpyzca	Pyrazine-2-carboxylic acid
RH	Relative humidity
RhB/6G	Rhodamine B/6G
RR	Recycling runs
H_2_SBA	4,4′-sulfonyldibenzoic acid
H_2_suc	Succinic acid
SDZ	Sulfadiazine
STZ	Sulfathiazole
tbb	1,4-bis(thiabendazole-1-yl)-2-butene
H_3_tca	Tricarballylic acid
H_3_tcpb	1,3,5-tris(4-carboxyphenoxy)benzene
H_2_tdc	Thiophenedicarboxylic acid
TEC	Tetracycline
5-*tert*-H_2_bipa	5-*tert*-butylisophthalic acid
tib	1,3,5-tris(1-imidazolyl)benzene
4-tkpvb	1,2,4,5-tetrakis(4-pyridylvinyl)benzene
H_2_tpca	2,3,5,6-tetrabromoterephtalic acid
TNP	2,4,6-trinitrophenol

## References

[1] World Health Organization 10 Chemicals of Public Health Concern. https://www.who.int/news-room/photo-story/photo-story-detail/10-chemicals-of-public-health-concern.

[2] International Agency for Research on Cancer (IARC) Agents Classifed by the IARC Monographs, Volume 1–131. https://monographs.iarc.who.int/agents-classified-by-the-iarc/.

[3] Sheikh Kulsum P.G.P., Khanam R., Das S., Nayak A.K., Tack F.M.G., Meers E., Vithanage M., Shahid M., Kumar A., Chakraborty S. (2023). A state-of-the-art review on cadmium uptake, toxicity, and tolerance in rice: From physiological response to remediation process. Environ. Res..

[4] Howard J.A., Kuznietsova H., Dziubenko N., Aigle A., Natuzzi M., Thomas E., Lysenko V., David L., Brichart T., Lux F. (2023). Combating lead and cadmium exposure with an orally administered chitosan-based chelating polymer. Sci. Rep..

[5] Saedi S., Watson S.E., Young J.L., Tan Y., Wintergerst K.A., Cai L. (2023). Does maternal low-dose cadmium exposure increase the risk of offspring to develop metabolic syndrome and/or type 2 diabetes?. Life Sci..

[6] Peana M., Pelucelli A., Chasapis C.T., Perlepes S.P., Bekiari V., Medici S., Zoroddu M.A. (2023). Biological Effects of Human Exposure to Environmental Cadmium. Biomolecules.

[7] Xiong L., Zhou B., Young J.L., Xu L., Wintergerst K., Cai L. (2022). Effects of whole-life exposure to low-dose cadmium with post-weaning high-fat diet on offspring testes in a male mouse model. Chem. Biol. Interact..

[8] Ruczaj A., Brzóska M.M. (2023). Environmental exposure of the general population to cadmium as a risk factor of the damage to the nervous system: A critical review of current data. J. Appl. Toxicol..

[9] Nordberg M., Nordberg G.F. (2022). Metallothionein and Cadmium Toxicology-Historical Review and Commentary. Biomolecules.

[10] de Frémont P., Adet N., Parmentier J., Xu X., Jacques B., Dagorne S. (2022). Cationic organometallic complexes of group 12 metals: A decade of progress toward the quest of novel Lewis acidic catalysts. Coord. Chem. Rev..

[11] Vasile Scaeteanu G., Maxim C., Badea M., Olar R. (2023). Zinc (II) Carboxylate Coordination Polymers with Versatile Applications. Molecules.

[12] Parmar B., Bisht K.K., Rachuri Y., Suresh E. (2020). Zn (II)/Cd (II) based mixed ligand coordination polymers as fluorosensors for aqueous phase detection of hazardous pollutants. Inorg. Chem. Front..

[13] Jeong A.R., Shin J.W., Jeong J.H., Jeoung S., Moon H.R., Kang S., Min K.S. (2020). Porous and Nonporous Coordination Polymers Induced by Pseudohalide Ions for Luminescence and Gas Sorption. Inorg. Chem..

[14] Erxleben A. (2003). Structures and properties of Zn (II) coordination polymers. Coord. Chem. Rev..

[15] Kreno L.E., Leong K., Farha O.K., Allendorf M., Van Duyne R.P., Hupp J.T. (2012). Metal–Organic Framework Materials as Chemical Sensors. Chem. Rev..

[16] Dutta A., Pan Y., Liu J.-Q., Kumar A. (2021). Multicomponent isoreticular metal–organic frameworks: Principles, current status and challenges. Coord. Chem. Rev..

[17] Ding M., Flaig R.W., Jiang H., Yaghi O.M. (2019). Carbon capture and conversion using metal–organic frameworks and MOF-based materials. Chem. Soc. Rev..

[18] Allendorf M.D., Stavila V., Witman M., Brozek C.K., Hendon C.H. (2021). What Lies beneath a Metal–Organic Framework Crystal Structure? New Design Principles from Unexpected Behaviors. J. Am. Chem. Soc..

[19] Li J.-R., Sculley J., Zhou H.-C. (2012). Metal–Organic Frameworks for Separations. Chem. Rev..

[20] Ghorai P., Hazra A., Mandal J., Malik S., Brandão P., Banerjee P., Saha A. (2023). Selective Low-Level Detection of a Perilous Nitroaromatic Compound Using Tailor-Made Cd (II)-Based Coordination Polymers: Study of Photophysical Properties and Effect of Functional Groups. Inorg. Chem..

[21] Duan C., Yu Y., Xiao J., Li Y., Yang P., Hu F., Xi H. (2021). Recent advancements in metal–organic frameworks for green applications. Green Energy Environ..

[22] Shivam, Megha R., Lakhani V., Vala S., Dharaskar S., Paluvai N.R., Sinha M.K., Jampa S.S. (2023). Removal of heavy metals and dyes from its aqueous solution utilizing metal organic Frameworks (MOFs): Review. Mater. Today Proc..

[23] Cao T., Peng Y., Liu T., Wang S., Dou J., Li Y., Zhou C., Li D., Bai J. (2014). Assembly of a series of d^10^ coordination polymers of pamoic acid through a mixed-ligand synthetic strategy: Syntheses, structures and fluorescence properties. CrystEngComm.

[24] Liu Q.-F., Liu W., Cao Y.-P., Dong Y.-L., Liu H.-M. (2016). Synthesis, structure, and luminescent property of a new Cd (II) coordination polymer with (4, 8)-connected topology. Inorg. Nano-Met. Chem..

[25] Li Z.-H., Zhang J., Qin Q.-P. (2020). Synthesis, structure and luminescence properties of a three-dimensional Cd (II) coordination polymer with (3, 7)-connected topology. J. Sulfur Chem..

[26] Xu M., Liang G., Wang S., Ma X., Liang G., Ni Q. (2020). Structural variability, topology and luminescent properties of three new cadmium (II) coordination polymers based on 4′,4′,4′-[(trimethylamino)]-tris[(1,1′-biphenyl)-2-carboxylate]. J. Mol. Struct..

[27] Narea P., Cisterna J., Cárdenas A., Amo-Ochoa P., Zamora F., Climent C., Alemany P., Conejeros S., Llanos J., Brito I. (2020). Crystallization induced enhanced emission in two new Zn (II) and Cd (II) supramolecular coordination complexes with the 1-(3,4-dimethylphenyl)-5-methyl-1H-1,2,3-triazole-4-carboxylate ligand. Polymers.

[28] Bai Y.-T., Zheng H., Tong K.-W., Feng S.-S., Zhu M.-L. (2021). Construction of a one-dimensional cadmium coordination polymer based on a triangle flexible multicarboxylate linker. Inorg. Nano-Met. Chem..

[29] Wang N., Long B.-F., Yin X.-H., Huang Z.-j., Mi Y., Hu F.-L., Young D.J. (2021). New structurally diverse photoactive cadmium coordination polymers. Dalton Trans..

[30] Lozovan V., Kravtsov V.C.H., Costriucova N.V., Siminel A.V., Kulikova O.V., Fonari M.S. (2022). Tunability in dimension, metal and ligand coordination modes and emission properties in Cd (II) and Zn (II) coordination networks based on 4,4′-(hydrazine-1,2-diyilidenebis (methanylylidene)) dibenzoic acid linker. J. Solid State Chem..

[31] Lin H., Deng X., Sun Y., Chen S., Zhou T. (2022). Effect of N-donor ancillary ligand on zinc/cadmium-organic arsonates: Structural analysis and photoluminescence. J. Solid State Chem..

[32] Cui S., Zhang W., Zhu B., Yuan N., Yu J., Sun Z., Li J., Zuo M. (2022). Two novel cadmium coordination polymers bearing viologen-derived ligand: Structure and photochromism properties. Inorg. Chim. Acta.

[33] Li Z.-Y., Chang H., Zhao J.-J., Zhang C., Wu D.-Q., Zhai B. (2023). Tunable structures and magnetic/optical properties of six Cd (II)-based coordination polymers by introducing different para- or dia-magnetic metal ions. J. Mol. Struct..

[34] Liu Y.-H., Liao T.-T., Lin S.-Y., Zhong S.-Y., Chen T.-R., Chen J.-D. (2023). Cd (II) and Zn (II) coordination polymers constructed from bis-pyridyl-bis-amide and dicarboxylic or tetracarboxylic acid: Synthesis, structures and luminescent properties. Inorg. Chim. Acta.

[35] He S.-S., Jiang T., Fu W.-W., Chen H., Li S., Zhou P., Shen J.-R., Liu Y., Chen M.-S. (2023). Six novel Zn (II)/Cd (II) coordination polymers based on a rigid tridentate imidazolyl terpyridine ligand fine-tuned by different carboxylates: Syntheses, structural diversities and luminescence properties. Polyhedron.

[36] Lozovan V., Kravtsov V.C.H., Chumakov Y.M., Costriucova N.V., Siminel N., Petuhov O., Vlase T., Vlase G., Barba A., Fonari M.S. (2023). Zn (II) and Cd (II) metal–organic frameworks with azine-functionalized pores: Crystal structures, photoluminescence, solvent exchange, and molecular simulations of carbon dioxide binding sites. Cryst. Growth Des..

[37] Zhang S., Lu K., Shen X., Ye Y., Sun G., Xu J., Han G. (2023). Two new alkali/cadmium bimetallic coordination polymers: Luminescent properties and cancer prevention by regulating the interaction between high mobility group protein B1 and immune cells. J. Clust. Sci..

[38] Liu L., Li J.-M., Zhang M.-D., Wang H.-J., Li Y., Zhang Z.-B., Zhao Z.-F., Xi Y., Huang Y.-Y., Xu J. (2023). Cd (II)/Mn (II)/Co (II)/Ni (II)/Zn (II) coordination polymers built from dicarboxylic acid/tetracarboxylic acid ligands: Their structural diversity and fluorescence properties. Polymers.

[39] Li Z., Bu J., Zhang R., Zhang C., Dongqing W., Zhai B. (2023). Two temperature-dependent 2D heterometallic Cd (II)–Dy (III) coordination polymers exhibiting slow magnetic relaxation and luminescence properties. J. Rare Earths.

[40] Wu Y.-Q., Wu F.F., Wang Z.-X., He X., Xing F.-F., Li M.-X. (2023). Syntheses, crystal structures, luminescent and magnetic properties of six 5,5′-(1,2-phenylenebis (methoxy)) diisophthalate coordination polymers. Inorg. Chim..

[41] Yang F., Chen J., Wang J., Liu J. (2023). Structure and photochromic properties of two cadmium coordination polymers derived from viologen ligands. Z. Anorg. Allg. Chem..

[42] Zhao Y., Zhao D.D., Liu H.L., Zhou W.Y., Li G. (2020). Imidazole Multi-Carboxylate-Based 2D Cd (II) MOF: Preparation, Crystal Structure, and Properties. Russ. J. Coord. Chem..

[43] Ye Y., Lin S., Wu X., Zhang X., Wang A. (2020). Solvothermal synthesis and luminescence properties of a novel Cd(II) coordination polymer containing 3-pyridinecarboxylate and phen ligands and [TaF_6_]^−^ anion. J. Mol. Struct..

[44] Xu B., Yao W., Yu X., Fedin V.P., Gao E. (2023). Cadmium (II)-Organic Coordination Polymer Containing Carboxyl Groups: Solvothermal Synthesis, Structure, and Properties. Russ. J. Coord. Chem..

[45] Liu G., Han S., Gao Y., Xu N., Wang X., Chen B. (2020). Multifunctional fluorescence responses of phenyl-amide-bridged d^10^ coordination polymers structurally regulated by dicarboxylates and metal ions. CrystEngComm.

[46] Song X., Dong W., Hou X., Zhao Q., Zhang Z., Ren Y. (2023). The high fluorescence sensitivity property and quenching mechanism of one-dimensional Cd-HCIA-1 sensor for nitrobenzene. Phys. Chem. Chem. Phys..

[47] Zhao Y.-Y., Chen L., Xu Z., Zhu C.-Y., Li P., Gao W., Li J.-Y., Zhang X.-M. (2023). Microporous Cd-MOF as multifunctional material for rapid and visual luminescence sensing of Fe^3+^, MnO_4_^−^ and TNP in water and efficient CO_2_ catalytic conversion. Micropor. Mesopor. Mater..

[48] Meyers A.E., Randolph R.K., LaDuca R.L. (2017). Divergent topologies in luminescent zinc and cadmium substituted isophthalate coordination polymers constructed from long-spanning dipyridylamide ligand precursors. Inorg. Chim. Acta.

[49] Li B., Duan W.X., Liu S.S., Jin Y.-J., Wang L.-Y. (2023). Zinc (II) and cadmium (II) coordination polymers constructed from 5-(benzimidazole-1-yl) isophthalic acid ligand: Syntheses, structures and detection of antibiotics in aqueous medium. J. Clust. Sci..

[50] Zhao Y.Y., Zhou Y., Li R., Li B. (2023). Synthesis, characterization and efficient detection of antibiotics of two Cd (II)-based coordination polymers. J. Clust. Sci..

[51] Du Y., Ghosh M.K., Lu L., Wang J., Mohanty A., Ghorai T.K., Afzal M., Alarifi A. (2023). Synthesized and characterization of a new Cd (II)-based coordination polymer: Sensing activity and photocatalytic activity against antibiotic. Polyhedron.

[52] Li J., Yao S.-L., Zheng T.-F., Xu H., Li J.-Y., Peng Y., Chen J.-L., Liu S.-J., Wen H.-R. (2022). Turn-on and blue-shift fluorescence sensor toward l-histidine based on stable Cd (II) metal–organic framework with tetranuclear cluster units. Dalton Trans..

[53] Cai S.-L., Lu L., Wu W.-P., Wang J., Sun Y.-C., Ma A.-Q., Singh A., Kumar A. (2019). A new mixed ligand based Cd (II) 2D coordination polymer with functional sites: Photoluminiscence and photocatalytic properties. Inorg. Chim. Acta.

[54] Cai H., Li N., Li Y., An D.-M. (2020). New three-dimensional Zn (II)/Cd (II)-based coordination polymers as luminescent sensor for Cu^2+^. Inorg. Chim. Acta.

[55] Contejean Z.I., LaDuca R.L. (2018). Nitroaromatic detecting zinc and cadmium coordination polymers with methyl-substituted aliphatic dicarboxylate and 4,4′-dipyridylamine ligands and diverse topologies. J. Solid State Chem..

[56] Gong W.-J., Yao R., Li H.-X., Ren Z.-G., Zhang J.-G., Lang J.-P. (2017). 2017. Luminescent cadmium (II) coordination polymers of 1,2,4,5-tetrakis (4-pyridylvinyl) benzene used as efficient multi-responsive sensors for toxic metal ions in water. Dalton Trans..

[57] Karbalaee Hosseini A., Tadjarodi A. (2023). Luminescent Cd coordination polymer based on thiazole as a dual-responsive chemosensor for 4-nitroaniline and CrO_4_^2−^ in water. Sci. Rep..

[58] Kabak B., Kendüzler E. (2022). Synthesis, characterization and adsorption/sensing applications of novel cadmium (II) based coordination polymer. J. Environ. Chem. Eng..

[59] Li S., Lu L., Zhu M., Yuan C., Feng S. (2018). A bifunctional chemosensor for detection of volatile ketone or hexavalent chromate anions in aqueous solution based on a Cd (II) metal–organic framework. Sens. Actuators B Chem..

[60] Wang Y.-N., Xu H., Wang S.-D., Zhang M.-H., Wang Y.-T., Qiu Q.-C., Bai J.-T., Mo Y., Feng W.-Y., Yang Q.-F. (2023). Multifunctional Cd-CP for fluorescence sensing of Cr (VI), MnO_4_^−^, acetylacetone and ascorbic acid in aqueous solutions. Spectrochim. Acta A Mol. Biomol. Spectrosc..

[61] Wang Y.-N., Xu H., Wang S.-D., Mao R.-Y., Wen L.-M., Wang S.-Y., Liu L.-J., Sun Y., Lu S.-Q., Wang F. (2023). A water-stable dual-responsive Cd-CP for fluorometric recognition of hypochlorite and acetylacetone in aqueous media. Spectrochim. Acta A Mol. Biomol. Spectrosc..

[62] Yan X.-L., Cao X.-Q., Deng C.-R., Zheng T.-F., Yao S.-L., Liu S.-J. (2023). A highly stable and efficient benzothiadiazole-based fluorescence sensor for salicylaldehyde in aqueous solution. CrystEngComm.

[63] Li W., Zhao D., Lei N., Wen R., Li W., Dou M., Fan L. (2023). Luminiscence sensing and electrocatalytic redox performances of a new stable cadmium (II) coordination polymer. J. Solid State Chem..

[64] Zhang M.-L., Lu X.-Y., Ren Y.-X., Wang J.-J., Yang X.-G. (2022). Three Cd (II) coordination polymers containing phenylenediacetate isomers: Luminescence sensing and adsorption antibiotics performance in water. Dye. Pigm..

[65] Wu Y.-B., Ren L., Dong G.-Y. (2022). Syntheses, crystal structures, luminiscent sensing and photocatalytic properties of two 2D cadmium (II) coordination polymers constructed from mixed ligands. Inorg. Chim. Acta.

[66] Chang J.-L., Wu J.-F., Zhang J.-W., Cui K., Liu Z.-Q. (2024). Crystal structure and fluorescence-based sensor properties of a Metal-Organic Framework. Z. Naturforsch. B.

[67] Wang Z.-P., Wang Y., Li X.-Y., Jia L.-F., Yang A.-Z., Zhao W.-T., Jia Y., Yu B.-Y., Zhao H.-Q. (2024). Water-stable mixed-ligand Cd (II) metal–organic frameworks as bis-color excited fluorescent sensors for the detection of vitamins and pesticides in aqueous solutions. J. Mol. Struct..

[68] Liu T.-Y., Qu X.-L., Zhang Y., Yan B. (2021). A Stable Cd (II)-Based Metal–Organic Framework: Synthesis, Structure, and Its Eu^3+^ Functionalization for Ratiometric Sensing on the Biomarker 2-(2-Methoxyethoxy) Acetic Acid. Inorg. Chem..

[69] Jiang Q.-J., Chuang P.-M., Wu J.-Y. (2023). Fluorescence-Responsive Detection of Ag (I), Al (III), and Cr (III) Ions Using Cd (II) Based Pillared-Layer Frameworks. Int. J. Mol. Sci..

[70] Li D., Xu H.-Q., Jiao L., Jiang H.-L. (2019). Metal–Organic Frameworks for Catalysis: State-of-the-Art, Challenges, and Opportunities. EnergyChem.

[71] Liu S.-H., Zhang J.-W., Wang X., Wang L.-H., Wang Z.-H., Wei Y.-B. (2017). Synthesis, crystal structure and catalytic property of a new cadmium coordinarion polymer. J. Clust. Sci..

[72] Zhang J.-W., Cheng Y., Liu S.-H. (2019). Synthesis, crystal structure and catalytic property of a cadmium (II) coordination polymer derived from a zwitterionic carboxylate ligand. Inorg. Nano-Met. Chem..

[73] Choi I.H., Kim Y., Lee D.N., Huh S. (2016). Three-dimensional cobalt (II) and cadmium (II) MOFs containing 1,4-naphtalenedicarboxylate: Catalytic activity of Cd-MOF. Polyhedron.

[74] Cheng X., Guo L., Wang H., Gu J., Yang Y., Kirillova M., Kirillov A. (2022). Coordination polymers from biphenyl-dicarboxylate linkers: Synthesis, structural diversity, interpenetration, and catalytic properties. Inorg. Chem..

[75] Palomo C., Oiarbide M., Laso A. (2007). Recent advances in the catalytic asymmetric nitroaldol (Henry) reaction. Eur. J. Org. Chem..

[76] Dallesandro E., Collin H., Guimarães L.G., Valle M., Pliego J. (2017). Mechanism of the piperidine-catalyzed Knoevenagel condensation reaction in methanol: The role of iminium and enolate ions. J. Phys. Chem. B.

[77] Sefidabi F., Abbasi A., Mortazavi S.-S., Masteri-Farahani M. (2020). A new 2D cadmium coordination polymer based on hydroxyl-substituted benzendicarboxylic acid as an effective heterogeneous catalyst for Knoevenagel condensation. Appl. Organomet. Chem..

[78] Yi X.-C., Huang M.-X., Qi Y., Gao E.-Q. (2014). Synthesis, structure, luminescence and catalytic properties of cadmium (II) coordination polymers with 9*H*-carbazole-2,7-dicarboxylic acid. Dalton Trans..

[79] Ji Z.X., Li P.F. (2018). Crystal structure and catalytic activity of a novel Cd (II) coordination polymer formed by dicarboxylic ligand. Bull. Chem. React. Eng..

[80] Karmakar A., Paul A., Guedes da Silva M.F.C., Pombeiro A.J.L. (2023). Polyaromatic group embedded Cd (II)-coordination polymers for microwave-assisted solvent-free Strecker-type cyanation of acetals. Molecules.

[81] Khan S., Markad D., Mandal S. (2023). Two Zn (II)/Cd (II) coordination polymers as recyclable heterogeneous catalysts for an efficient room-temperature synthesis of α-aminonitriles via the solvent-free Strecker reaction. Inorg. Chem..

[82] Gutić S.J., Dobrota A.S., Fako E., Skorodumova N.V., López N., Pašti I.A. (2020). Hydrogen Evolution Reaction—From Single Crystal to Single Atom Catalysts. Catalysts.

[83] Etaiw S., El-Bendary M. (2018). Cd (II) supramolecular coordination polymer incorporating pyrazine-2-carboxylic acid: Crystal structure, spectral characteristics and catalytic activity. J. Lumin..

[84] Hao S.Y., Li Y.H., Zhu J., Cui G.H. (2018). Synthesis, structures, luminiscence and photocatalytic properties of two nanostructured cadmium (II) coordination polymers synthesized by sonochemical process. Ultrason. Sonochem..

[85] Zhao X.-X., Qin Z.-B., Li Y.-H., Cui G.-H. (2018). New Cd (II) and Zn (II) coordination polymers showing luminescent sensing for Fe (III) and photocatalytic degrading methylene blue. Polyhedron.

[86] Lu L., Wang J., Zhou Y., Sun Y., Wu X., Singh A., Kumar A. (2020). Two new coordination polymers driven by polycarboxylate and N-donor spacers: Photocatalytic performance and theoretical analysis. Inorg. Chim. Acta.

[87] Wang J., Lu L., He J.-R., Wu W.-P., Gong C., Fang L., Pan Y., Singh A.K., Kumar A. (2019). Photocatalytic performances of two new Cd (II) and Zn (II)-based coordination polymers. J. Mol. Struct..

[88] Zhang X., Blatov V., Zhao Y.-Q., Hao Z.-C., Cui G.-H. (2016). Synthesis, structures, and properties of cadmium (II) and nickel (II) coordination polymers based on a 4,4′-biphenyl-containing ligand and aliphatic carboxylic acids. Z. Anorg. Allg. Chem..

[89] Feng Z.-Q., Yang X.-L., Ye Y.-F. (2014). Synthesis, crystal structures, luminescence, biological and catalytic properties of two d^10^ metal-organic coordination polymers constructed from mixed ligands. J. Inorg. Organomet. Polym. Mater..

[90] Muslim M., Ahmad M., Alam M.J., Ahmad S. (2023). Experimental and density functional theory investigation on one- and two-dimensional coordination polymers and their ZnO-doped nanocomposite materials for wastewater remediation. Sep. Purif. Technol..

[91] Zheng M., Chen J., Zhang L., Cheng Y., Lu C., Liu Y., Singh A., Trivedi M., Kumar A., Liu J. (2022). Metal organic frameworks as efficient adsorbents for drugs from wastewater. Mater. Today Commun..

[92] Agarwal R., Mukherjee S. (2016). One dimensional coordination polymers of Cd (II) and Zn (II): Synthesis, structure, polar packing through strong inter-chain hydrogen bonding and gas adsorption studies. Polyhedron.

[93] Haque F., Halder A., Ghosh S., Ghoshal D. (2019). Five coordination polymers of Cd (II) and Co(II) using 3,3′-azobispyridine and different carboxylates: Synthesis, structures and adsorption properties. Polyhedron.

[94] Nagarkar S., Chaudhari A., Ghosh S. (2012). Selective CO_2_ adsorption in a robust and water-stable porous coordination polymer with a new network topology. Inorg. Chem..

[95] Somnath, Tyagi L., Lama R., Siddiqui K.A. (2021). Gas sorption and luminiscence properties of activated forms of a Cd (II)-coordination polymer. J. Coord. Chem..

[96] Hua J., Wang M., Zhang D., Pei X., Zhao X., Ma X. (2022). A three-dimensional cadmium mixed ligands coordination polymer with CO_2_ adsorption ability. J. Struct. Chem..

[97] Sharma M., Senkovska I., Kaskel S., Bharadwaj P. (2011). Three-dimensional porous Cd (II) coordination polymer with large one-dimensional hexagonal channels: High pressure CH_4_ and H_2_ adsorption studies. Inorg. Chem..

[98] Leroux M., Mercier N., Allain M., Dul M.-C., Dittmer J., Kassiba A.H., Bellat J.-P., Weber G., Bezverkhyy I. (2016). Porous coordination polymer based on bipyridinium carboxylate linkers with high and reversible ammonia uptake. Inorg. Chem..

[99] Naskar K., Dey A., Maity S., Bhunia M., Ray P.P., Sinha C. (2019). Novel porous polycatenated iodo-cadmium coordination polymer for iodine sorption and electrical conductivity measurement. Cryst. Growth Des..

[100] Zhang T., Lin S., Yan T., Li B., Liang Y., Liu D., He Y. (2023). Integrating self-partitioned pore space and amine functionality into an aromatic-rich coordination framework with Ph stability for effective purification of C2 hydrocarbons. Inorg. Chem..

[101] Burlak P.V., Samsonenko D.G., Kovalenko K.A., Fedin V.P. (2023). Series of Cadmium–Organic Frameworks Based on Mixed Flexible and Rigid Ligands: Single-Crystal-to-Single-Crystal Transformations, Sorption, and Luminescence Properties. Inorg. Chem..

[102] Al’Abri A.M., Sharhan O., Halim S.N.A., Bakar N.K.A., Sherino B., Kamboh M.A., Nodeh H.R., Mohamad S. (2022). Effect of framework metal ions of analogous magnetic porous coordination polymers on adsorption of cationic and anionic dyes from aqueous solution. Chem. Pap..

[103] Sezer G.G., Arici M., Erucar I., Yeșilel O.Z., Özel H.U., Gemici B.T., Erer H. (2017). Zinc (II) and cadmium (II) coordination polymers containing phenylenediacetate and 4,4′-azobis (pyridine) ligands: Syntheses, structures, dye adsorption properties and molecular dynamics simulations. J. Solid State Chem..

[104] Ahmed A., Ali A., Ahmed M., Parida K., Ahmad M., Ahmad A. (2021). Construction and topological studies of a three dimensional (3D) coordination polymer showing selective adsorption of aromatic hazardous dyes. Sep. Purif. Technol..

[105] Gaur R. (2019). Selective anionic dye adsorption, topology and luminescence study of structurally diverse cadmium (II) coordination polymers. Inorg. Chem. Front..

[106] Karmakar A., Paul A., Santos P.M.R., Santos I.R.M., Guedes da Silva M.F.C., Pombeiro A.J.L. (2023). Novel anthracene and pyrene containing Cd (II)-based coordination polymers for adsorptive removal of toxic dyes from aqueous medium. Colloids Surf. A Physicochem. Eng. Asp..

[107] Luo B., Yu D., Huo J. (2020). Polynuclear Cd (II) coordination polymer with unique configuration for chromium pollutants removal. J. Solid State Chem..

[108] Ma D.-Y., Guo H.-F., Qin L., Li Y., Ruan Q.-T., Huang Y.-W., Xu J. (2014). Construction of a new 2D cadmium (II) coordination polymer based on N- and O-donor ligands: Synthesis, luminiscence and biological activities. J. Chem. Crystallogr..

[109] Liu H.-M., Shang X.-N. (2022). Two new Cd (II)/Zn (II) cordination polymers: Luminiscence properties and synergistic treatment activity with ultrasound therapy on uterine fibroids. Des. Monomers Polym..

[110] Soltani S., Akhbari K., White J. (2021). Effect of structural features on the stability and bactericidal potential of two cadmium coordination polymers. CrystEngComm.

[111] Balendra, Sanyukta, Mahboob A., Sevi M. (2023). Cadmium-based coordination polymers (CPs) constructed from two different V-shaped dicarboxylate ligands: Synthesis, structure and dielectric properties. Inorg. Chem. Commun..

[112] Liu Y.-R., Chen Y.-Y., Jing Y.-F., Xie L.-X., Li G. (2022). High water-assisted proton conductivities of two cadmium (II) complexes constructed from zwitterionic ligands. Inorg. Chem..

[113] Cheng Q., Qin L., Zhou J., Lin J., Lin X., Zhang G., Cai Y. (2019). Four new Zn (II) and Cd (II) coordination polymers using two amide-like aromatic multi-carboxylate ligands: Synthesis, structures and lithium-selenium batteries application. RSC Adv..

[114] Najafi E., Janghouri M., Hashemzadeh A., Ng S.W. (2023). Mixed ligand Cd (II) coordination architectures based on bulky anthracene-9-carboxylate ligand: Crystal structures and optical properties. Inorg. Chim. Acta.

[115] Lu Y.F., He Y.-H., Liang J.-B., Jin Q., Ou Y.-C., Wu J.-Z. (2022). First cadmium coordination compound as an efficient floculant for Congo Red. Inorg. Chem. Commun..

[116] Al-Fakeh M.S., Alsaedi R.O., Amiri N., Allazzam G.A. (2022). Synthesis, Characterization, and Antimicrobial of MnO and CdO Nanoparticles by Using a Calcination Method. Coatings.

[117] Payehghadr M., Morsali A. (2013). Thermolysis preparation of cadmium (II) oxide nanoparticles from a new three-dimensional cadmium (II) supramolecular compound. J. Struct. Chem..

[118] Ramazani M., Morsali A. (2011). Sonochemical syntheses of a new nano-plate cadmium (II) coordination polymer as a precursor for the synthesis of cadmium (II) oxide nanoparticles. Ultrason. Sonochem..

